# Supplementary material for: Novel Substituted Thiophenes and Sulf-Polyacetylene Ester from Echinops ritro L
Source: Molecules. 2019 Feb 22;24(4):805. doi: 10.3390/molecules24040805 (PMC6413031; doi:10.3390/molecules24040805)
Supplement: Supplementary file 1 [file molecules-24-00805-s001.pdf]

Supplementary File

Substituted thiophenes and one sulf-polyacetylene ester compounds from *Echinops ritro* L. and their antimicrobial activities

Liang-Bo Li[1], Guang-Da Xiao[1], Wei Xiang[1], Xing Yang[1], Ke-Xin Cao[1], Rong-Shao Huang[1*]

[1] Agricultural College of Guangxi University, Nanning 530004, P. R. China; llb100@126.com (L.-B.L); xgd0104@163.com (G.-D.X.); victorxiang@st.gxu.edu.cn (W.X.); Hdyx0000@163.com (X.Y); 18991697639@163.com (K.-X.C.)

[*]Correspondence: hrs17252@gxu.edu.cn; Tel./Fax: +86-771-323-5612

Contents

Figure1. HR-ESI-MS of Compound 1..S3-S5

Figure 2. IR of Compound 1..S6

Figure 3. ^{1}H NMR (500MHz, CD_3OD) of Compound 1...S7

Figure4. ^{13}C NMR (125MHz, CD_3OD) of Compound 1...S8

Figure 5. 2D NMR (HSQC) of Compound 1..S9

Figure 6. 2D NMR (HMBC) of Compound 1..S10

Figure 7. 2D NMR (^{1}H-^{1}H COSY) of Compound 1..S11

Figure8. HR-ESI-MS of Compound 2..S12-S14

Figure 9. IR of Compound 2..S15

Figure10. ^{1}H NMR (500MHz, CD_3OD) of Compound 2..S16

Figure11. ^{13}C NMR (125MHz, CD_3OD) of Compound 2...S17

Figure 12. 2D NMR (HSQC) of Compound 2..S18

Figure 13. 2D NMR (HMBC) of Compound 2...S19

Figure14. 2D NMR (^{1}H-^{1}H COSY) of Compound 2..S20

Figure15. HR-ESI-MS of Compound 3..S21-S23

Figure 16. IR of Compound 3..S24

Figure 17. ^{1}H NMR (500MHz, CD_3OD) of Compound 3..S25

Figure18. ^{13}C NMR (125MHz, CD_3OD) of Compound 3..S26

Figure19. HR-ESI-MS of Compound 4..S27-S28

Figure 20. IR of Compound 4..S29

Figure 21. ^{1}H NMR (500MHz, CD_3OD) of Compound 4..S30

Figure22. ^{13}C NMR (125MHz, CD_3OD) of Compound 4..S31

Figure 23. 2D NMR (HSQC) of Compound 4..S32

Figure 24. 2D NMR (HMBC) of Compound 4...S33

Figure 25. 2D NMR (^{1}H-^{1}H COSY) of Compound 4...S34

Figure 26. 2D NMR (ROESY) of Compound 4...S35

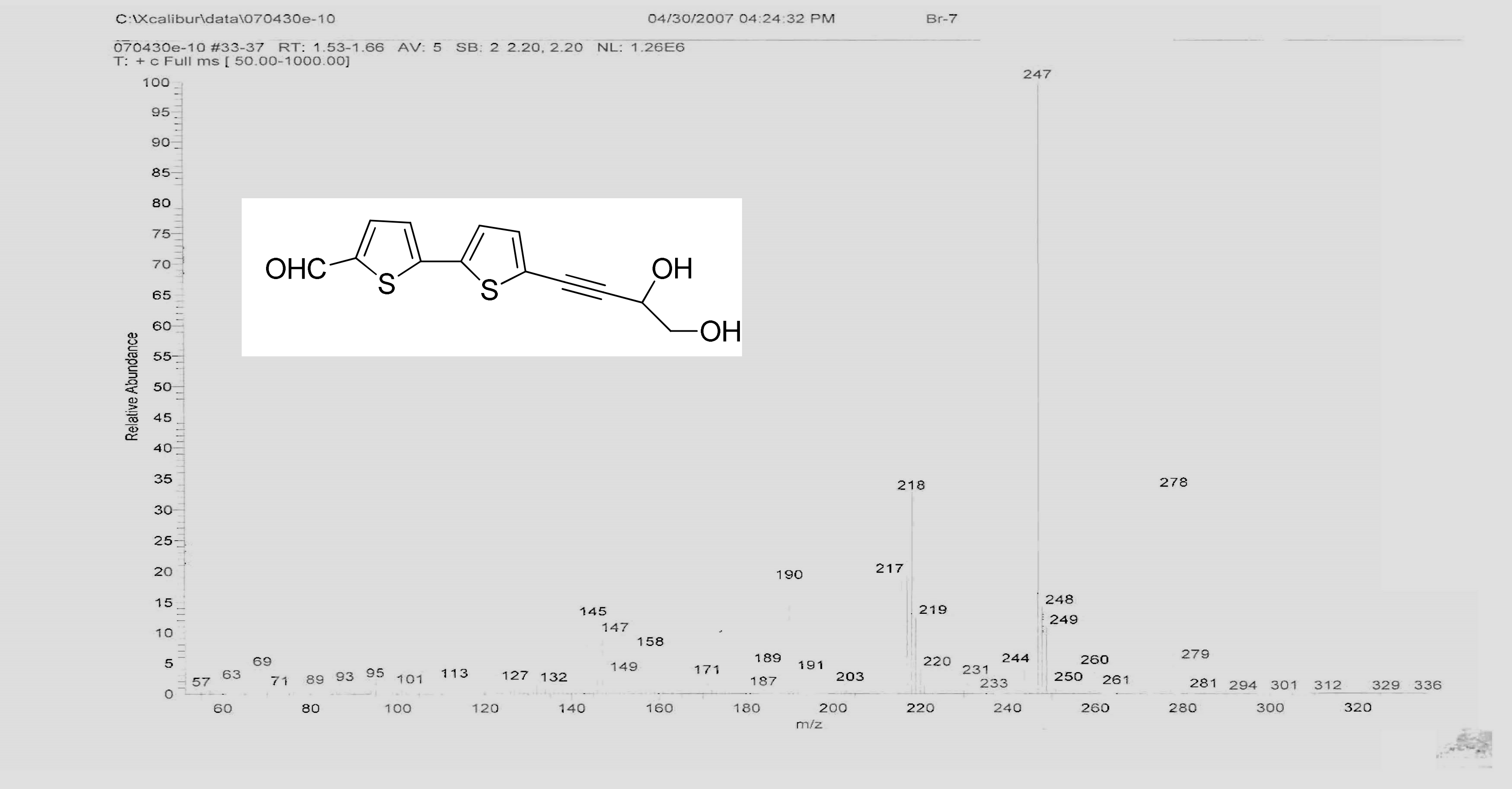
C:\Xcalibur\data\070430e-10
04/30/2007 04:24:32 PM
Br-7
070430e-10 #33-37 RT: 1.53-1.66 AV: 5 SB: 2 2.20, 2.20 NL: 1.26E6
T: + c Full ms [50.00-1000.00]
Relative Abundance
m/z
OHC
S
S
OH
OH
247
218
278
217
190
248
219
249
145
147
158
279

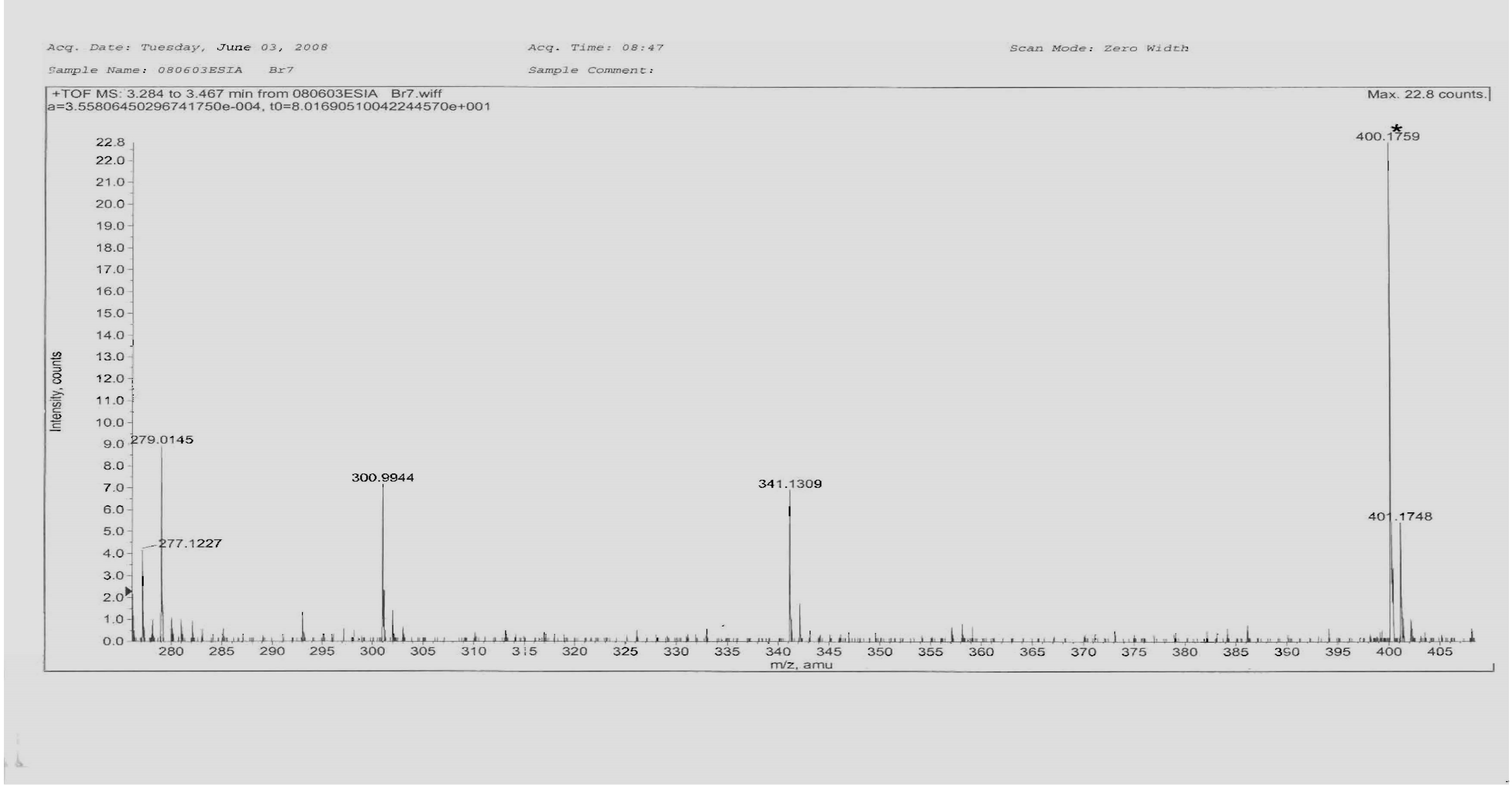

Acq. Date: Tuesday, June 03, 2008
Acq. Time: 08:47
Scan Mode: Zero Width
Sample Name: 080603ESIA Br7
Sample Comment:
+TOF MS: 3.284 to 3.467 min from 080603ESIA Br7.wiff
a=3.55806450296741750e-004, t0=8.01690510042244570e+001
Max. 22.8 counts.
Intensity, counts
m/z, amu
400.1759
401.1748
341.1309
300.9944
279.0145
277.1227

Acq. Date: Tuesday, June 03, 2008 Acq. Time: 08:47 Scan Mode: Zero Width

Sample Name: 080603ESIA Br7 Sample Comment:

Elemental composition calculator

Target m/z: +279.0145 amu
Tolerance: +10.0000 ppm
Result type: Elemental
Max num of results: 1000
Min DBE: -5.0000 Max DBE: +60.0000
Electron state: OddAndEven
Num of charges: 0
Add water: N/A
Add proton: N/A
File Name: 080603ESIA Br7.wiff

	Elements	Min Number	Max Number
1	Br	0	0
2	C	0	100
3	Cl	0	0
4	F	0	0
5	H	0	400
6	I	0	0
7	N	0	0
8	Na	0	0
9	O	0	4
10	S	2	2

Acq. Date: Tuesday, June 03, 2008 Acq. Time: 08:47 Scan Mode: Zero Width

Sample Name: 080603ESIA Br7 Sample Comment:

	Formula	Calculated m/z (amu)	mDa Error	PPM Error	DBE
1	C13 H11 O3 S2	279.0149	-0.4629	-1.6592	8.5

Figure 1 HR-ESI-MS of Compound **1**

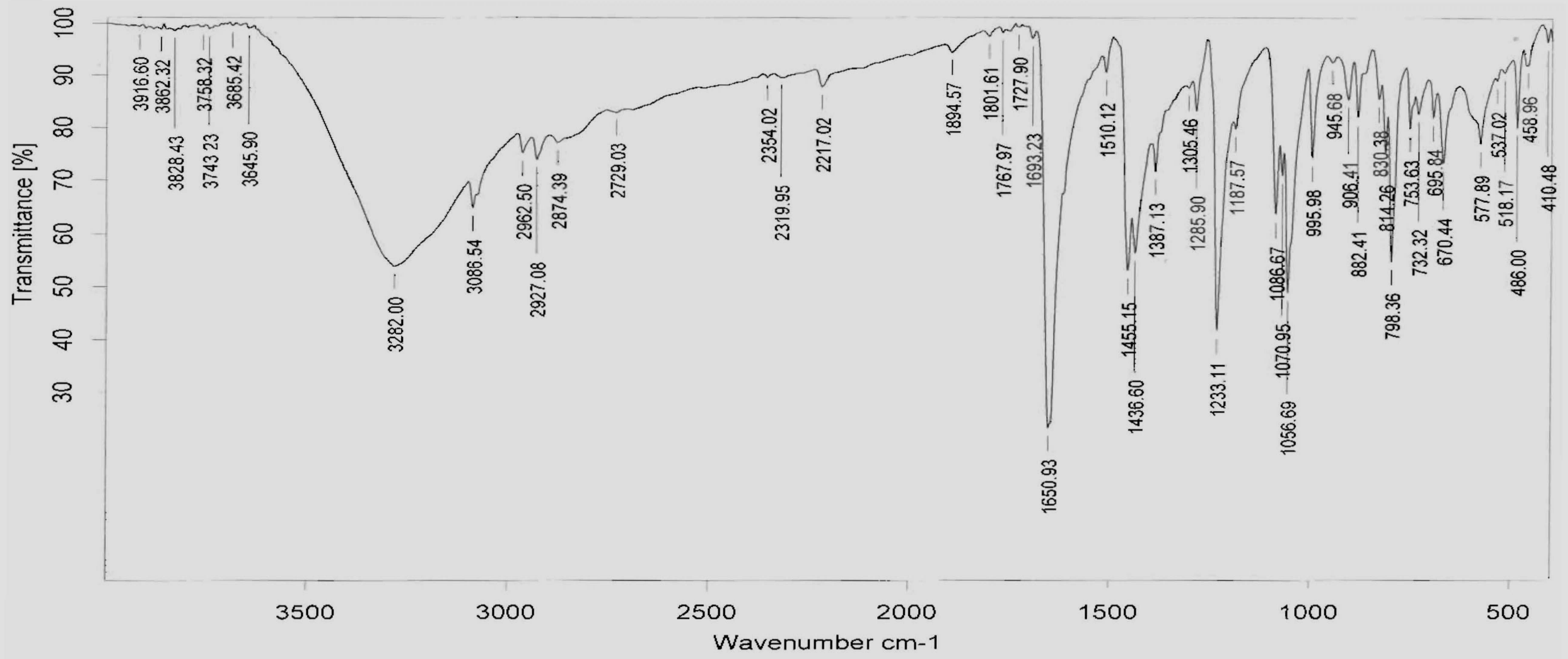

Sample : Br7	Frequency Range : 399.271 - 3996.57	Measured on : 03/02/2010	
Technique : KBr压片	Resolution : 4	Instrument : Tensor27	Sample Scans : 16
Customer : 100203IR8	Zerofilling : 2	Acquisition : Double Sided,For	

Figure 2 IR of Compound **1**

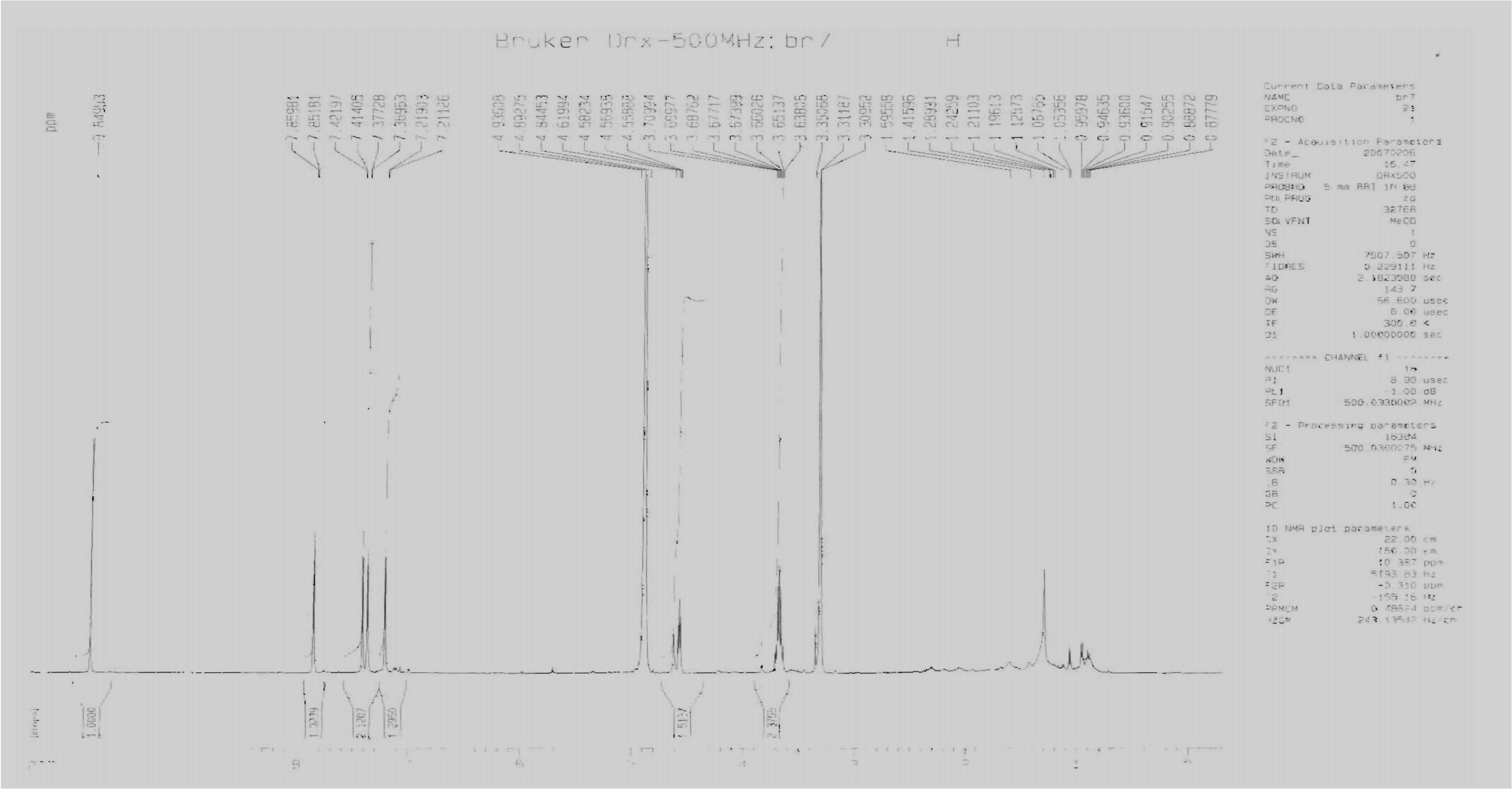

Figure 3 ^{1}H NMR (500 MHz, CD_3OD) of Compound **1**

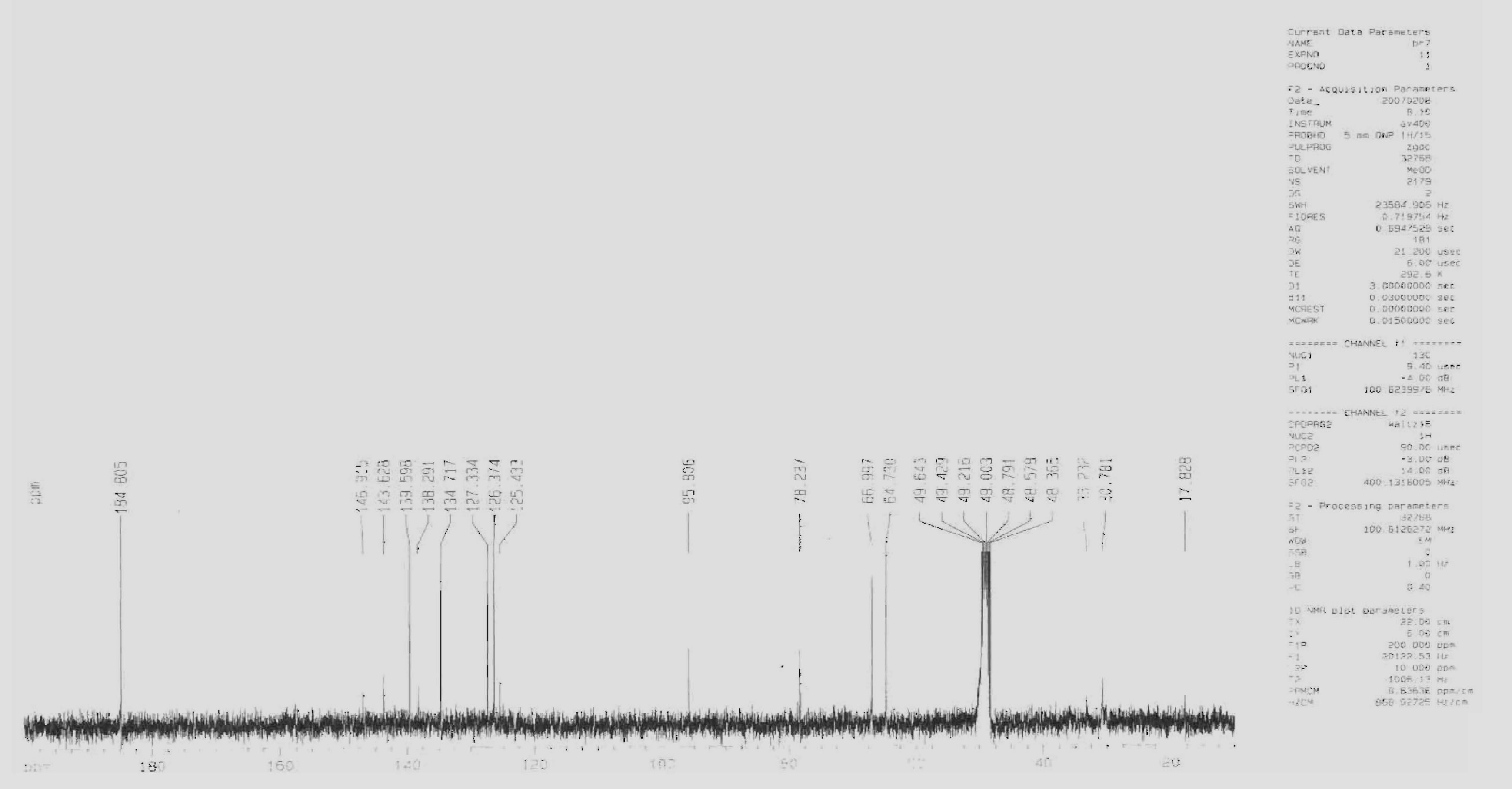

Figure 4 ^{13}C NMR (125 MHz, CD_3OD) of Compound **1**

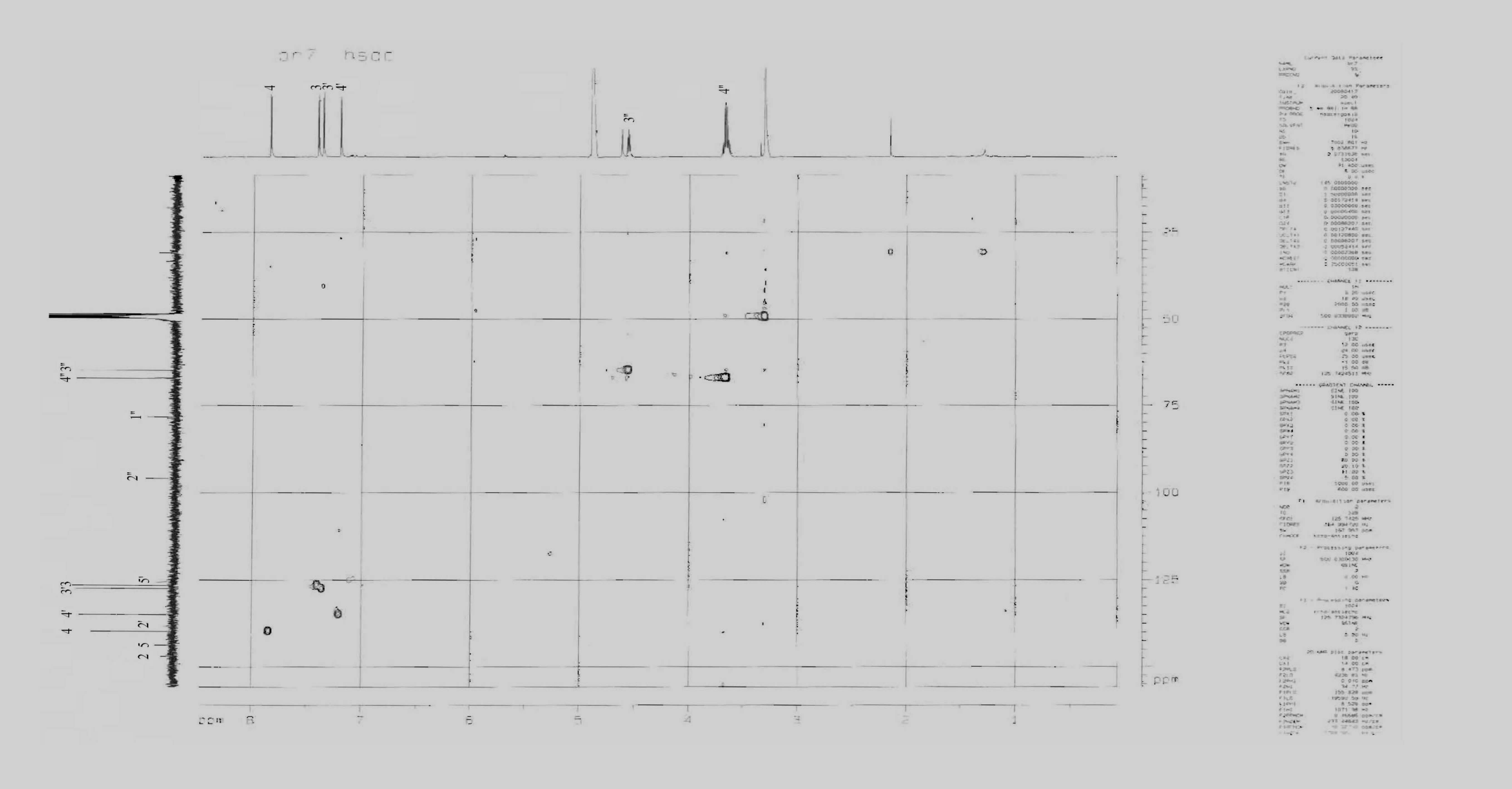

Figure 5 2D NMR (HSQC) of Compound **1**

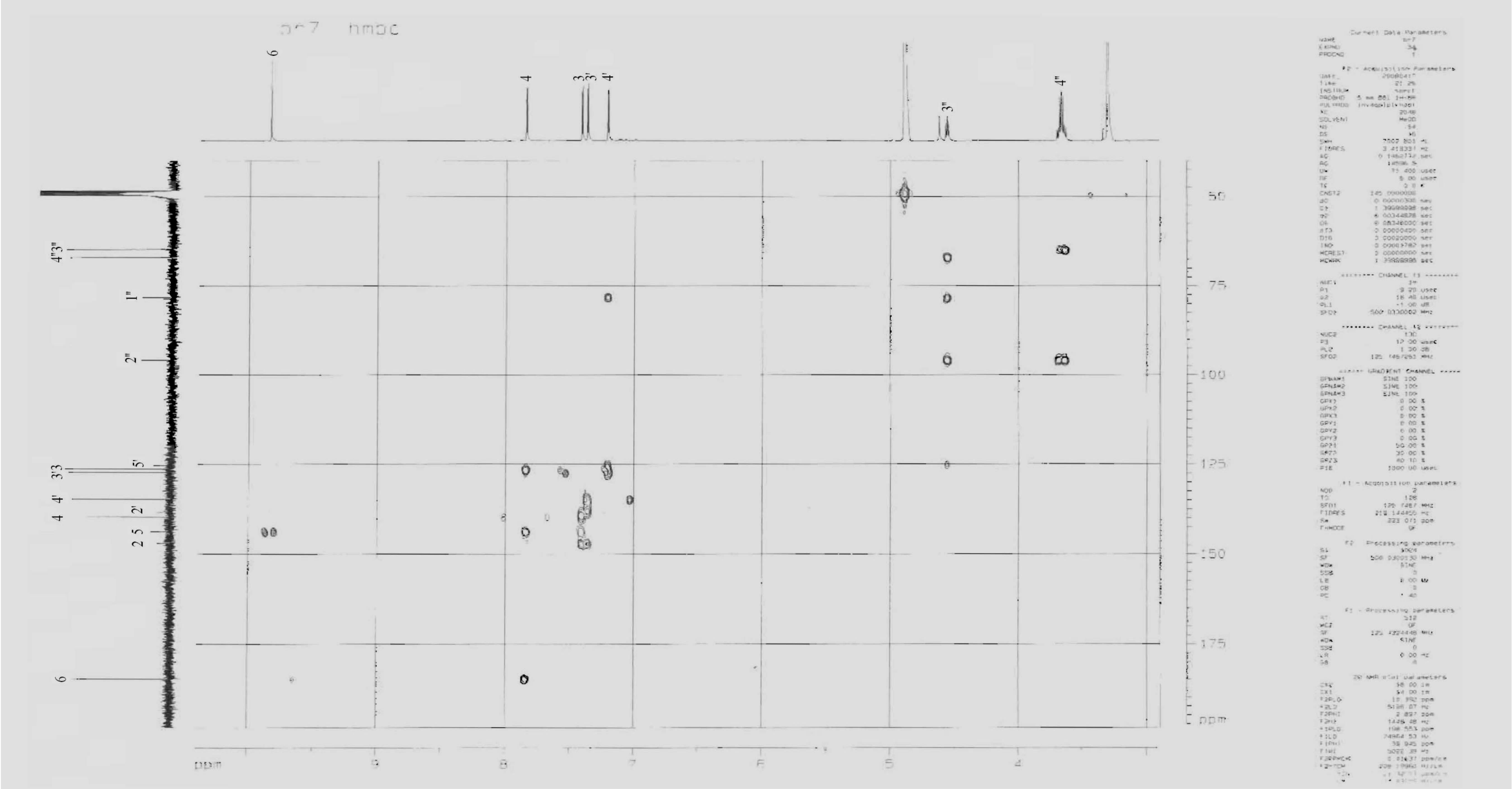

Figure 6 2D NMR (HMBC) of Compound **1**

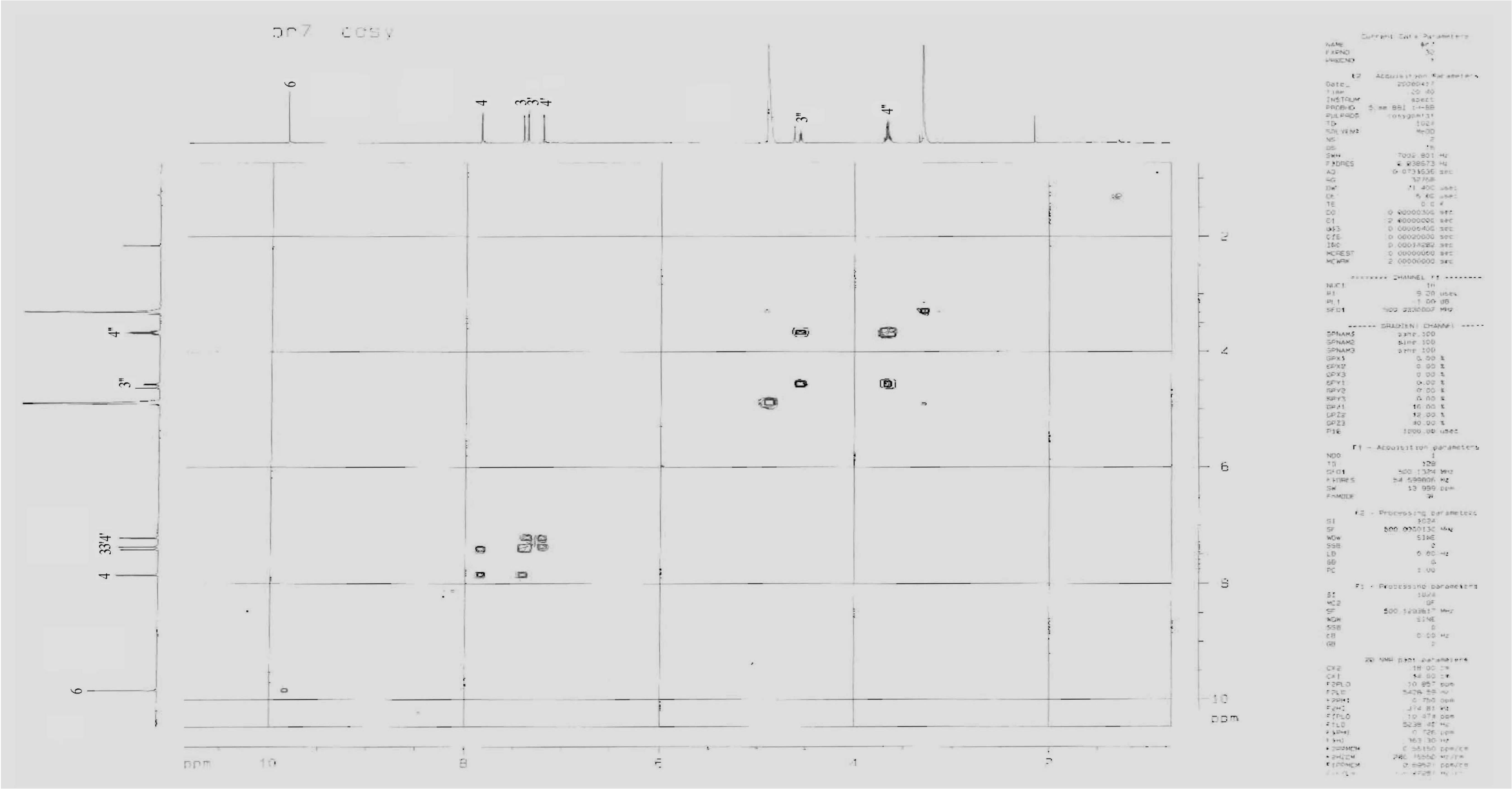

Figure 7 2D NMR (^1H-^1H COSY) of Compound **1**

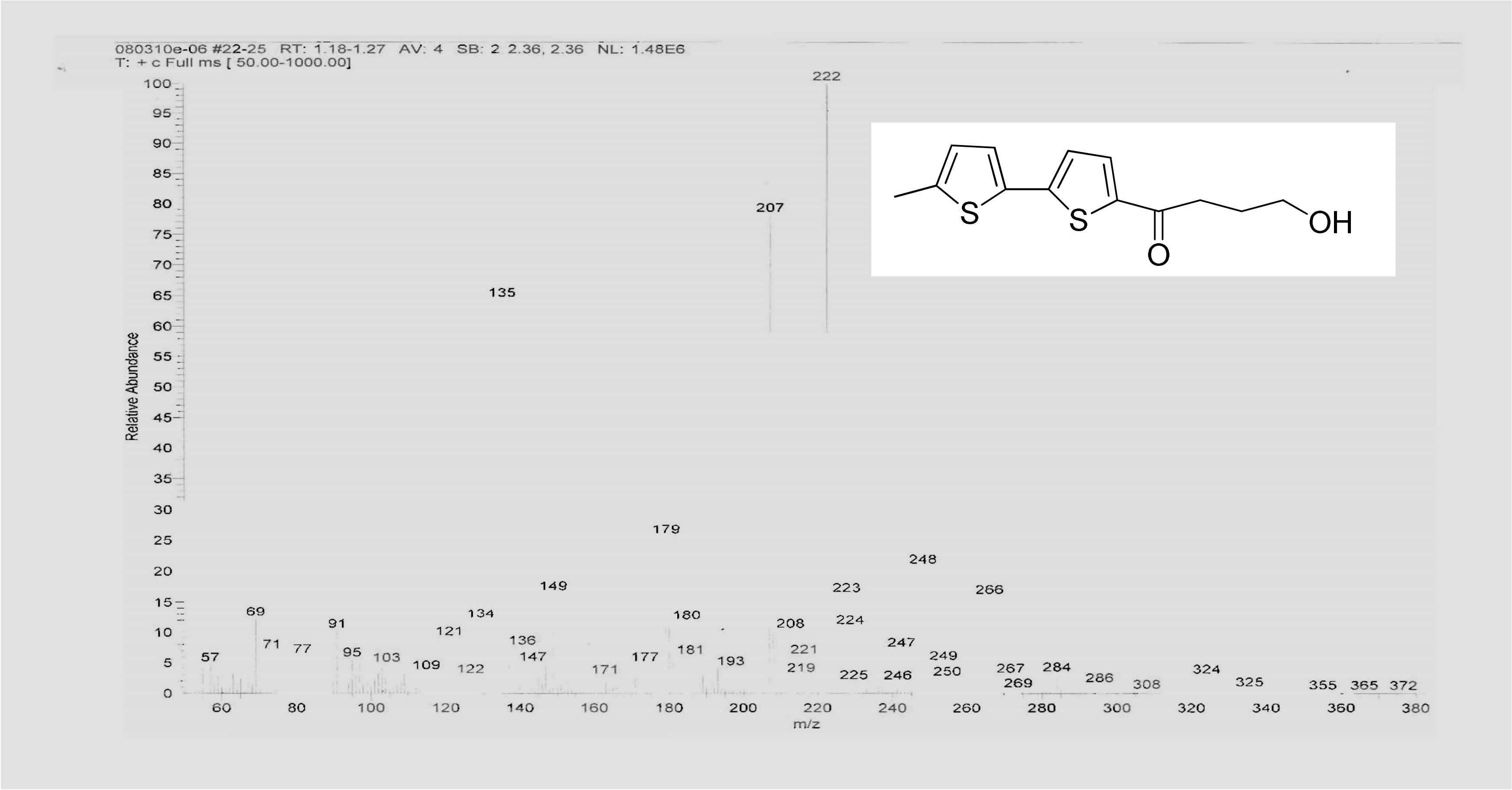
080310e-06 #22-25 RT: 1.18-1.27 AV: 4 SB: 2 2.36, 2.36 NL: 1.48E6
T: + c Full ms [50.00-1000.00]
Relative Abundance
m/z
S
S
O
OH

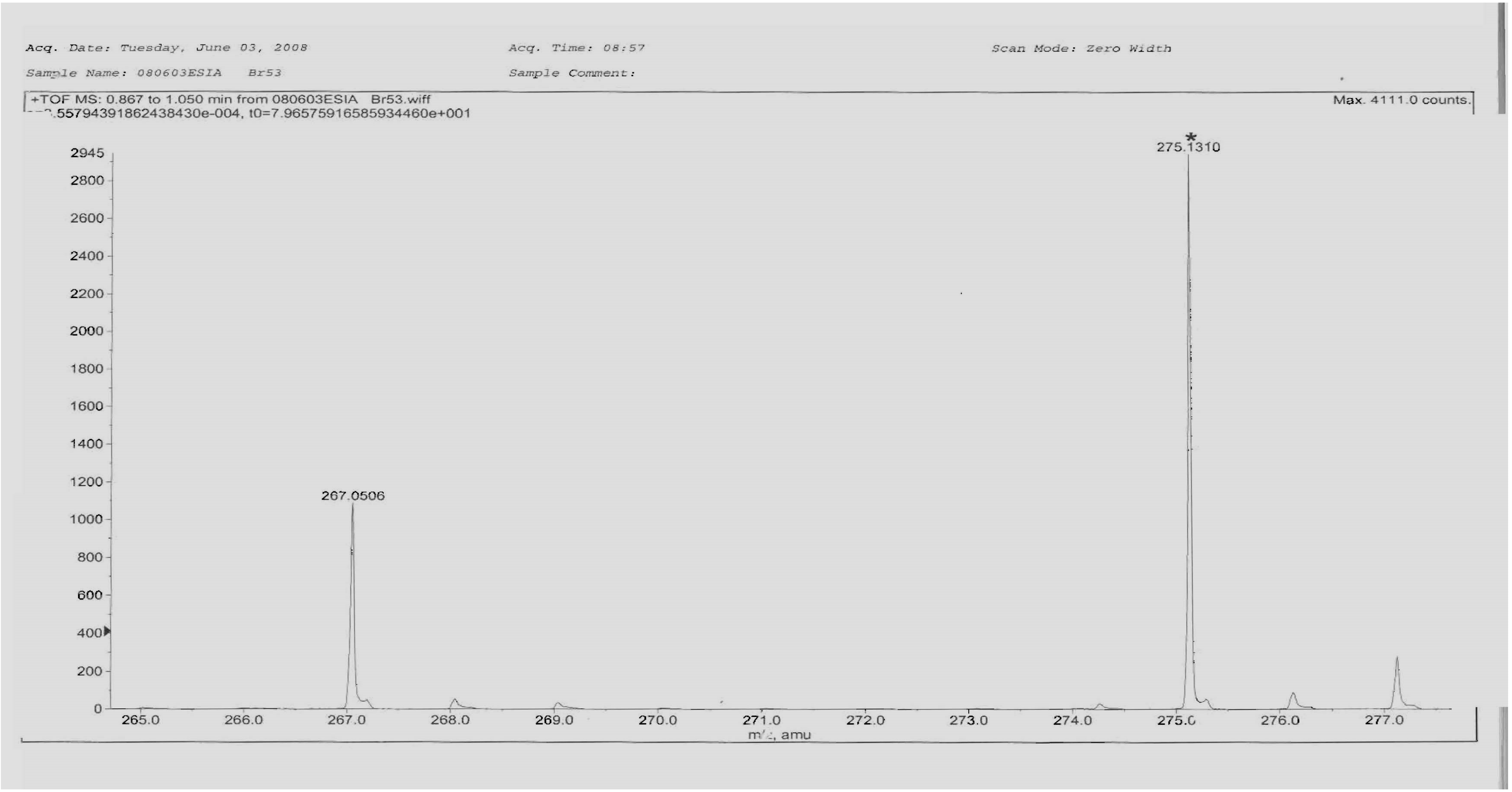
Acq. Date: Tuesday, June 03, 2008
Acq. Time: 08:57
Scan Mode: Zero Width
Sample Name: 080603ESIA Br53
Sample Comment:
+TOF MS: 0.867 to 1.050 min from 080603ESIA Br53.wiff
.55794391862438430e-004, t0=7.96575916585934460e+001
Max. 4111.0 counts.
275.1310
267.0506
2945
2800
2600
2400
2200
2000
1800
1600
1400
1200
1000
800
600
400
200
0
265.0
266.0
267.0
268.0
269.0
270.0
271.0
272.0
273.0
274.0
276.0
277.0
amu

Acq. Date: Tuesday, June 03, 2008 Acq. Time: 08:57 Scan Mode: Zero Width
Sample Name: 080603ESIA Br53 Sample Comment:

Elemental composition calculator

Target m/z: +267.0506 amu
Tolerance: +10.0000 ppm
Result type: Elemental
Max num of results: 1000
Min DBE: -5.0000 Max DBE: +60.0000
Electron state: OddAndEven
Num of charges: 0
Add water: N/A
Add proton: N/A
File Name: 080603ESIA Br53.wiff

	Elements	Min Number	Max Number
1	Br	0	0
2	C	0	100
3	Cl	0	0
4	F	0	0
5	H	0	400
6	I	0	0
7	N	0	0
8	Na	0	0
9	O	0	3
10	S	2	2

Acq. Date: Tuesday, June 03, 2008 Acq. Time: 08:57 Scan Mode: Zero Width
Sample Name: 080603ESIA Br53 Sample Comment:

	Formula	Calculated m/z (amu)	mDa Error	PPM Error	DBE
1	C13 H15 O2 S2	267.0513	-0.7484	-2.8027	6.5

Figure 8 HR-ESI-MS of Compound **2**

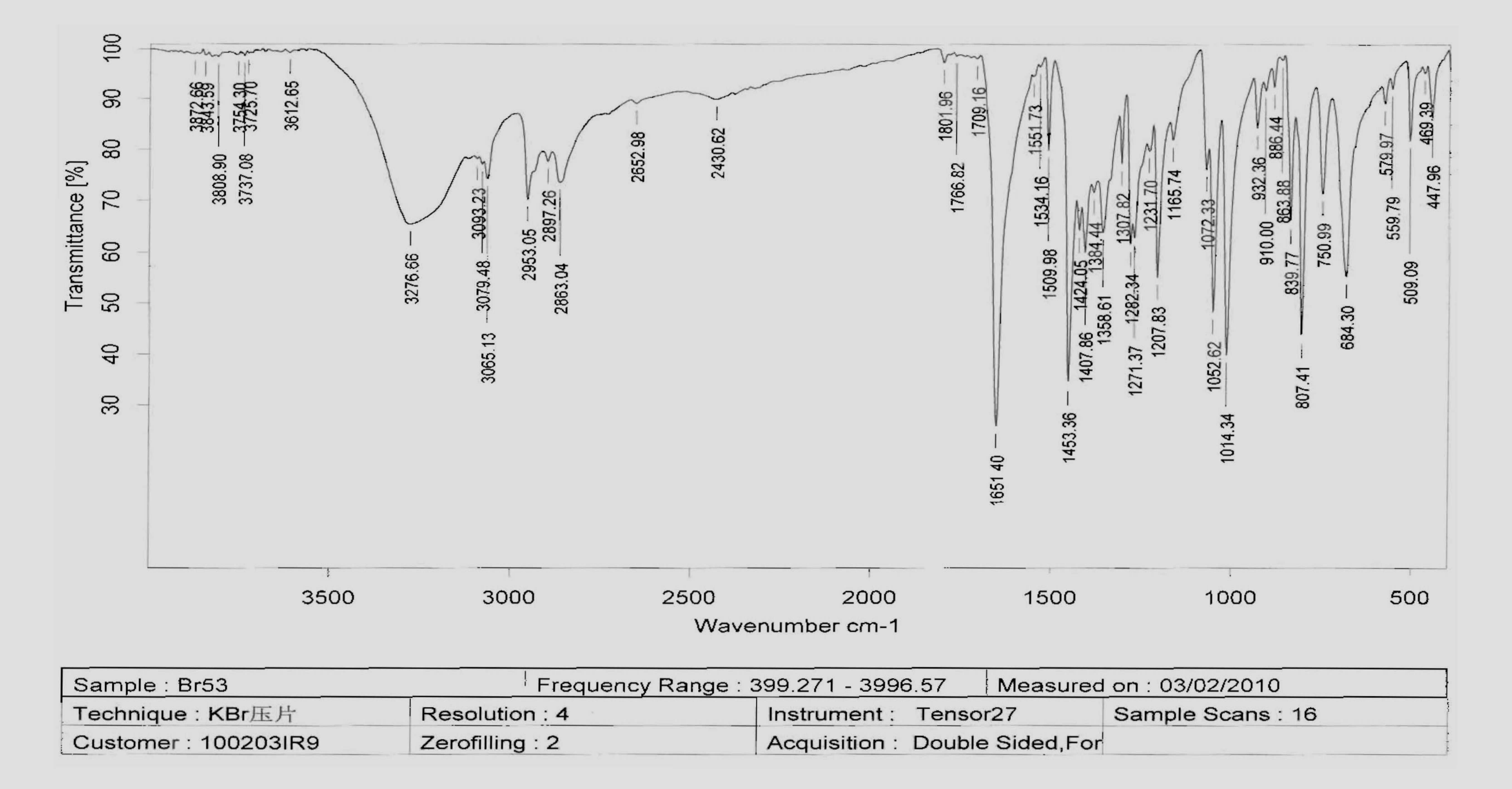

Figure 9 IR of Compound **2**

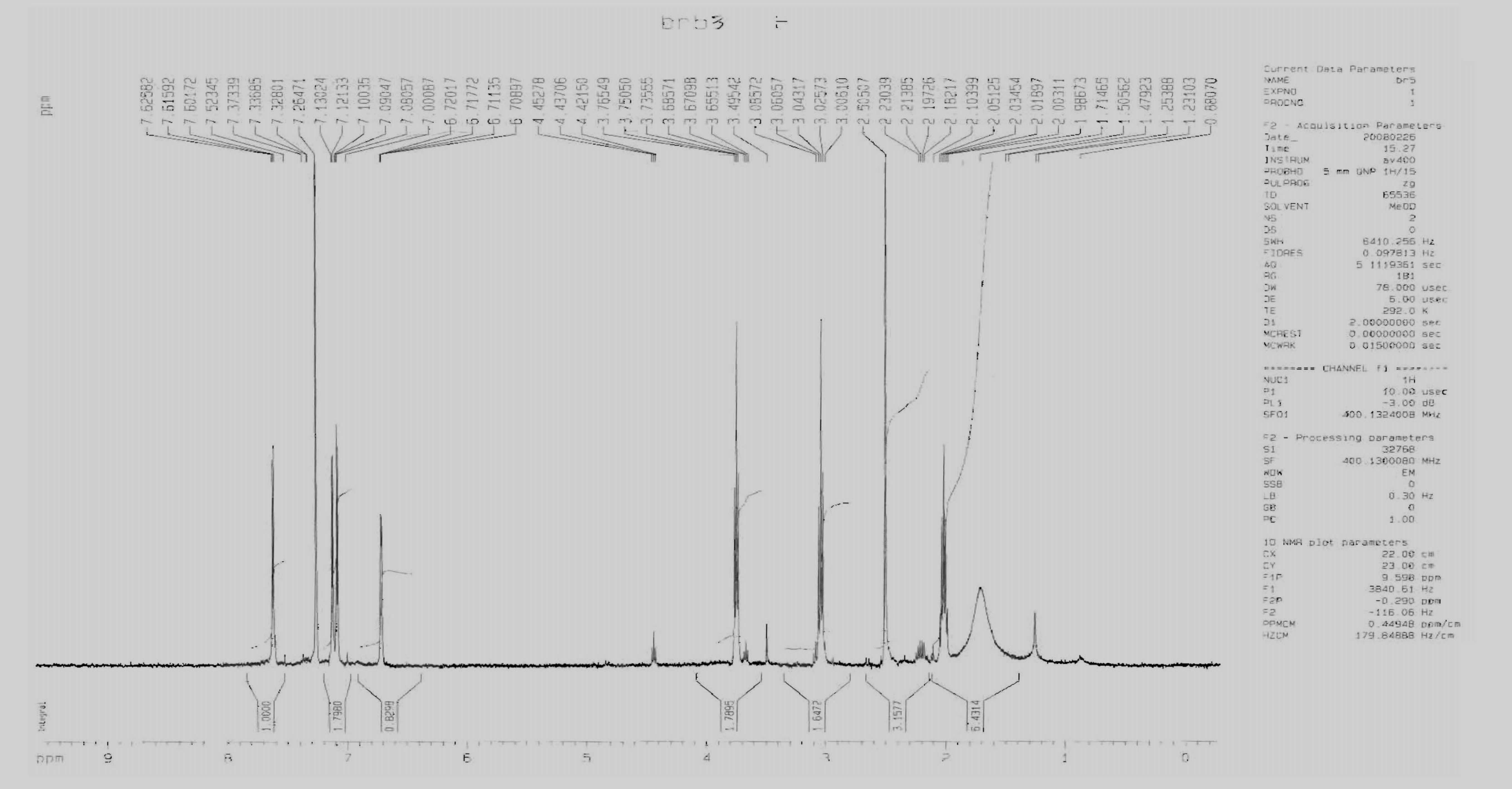

Figure 10 ^{1}H NMR (500 MHz, $CDCl_3$) of Compound **2**

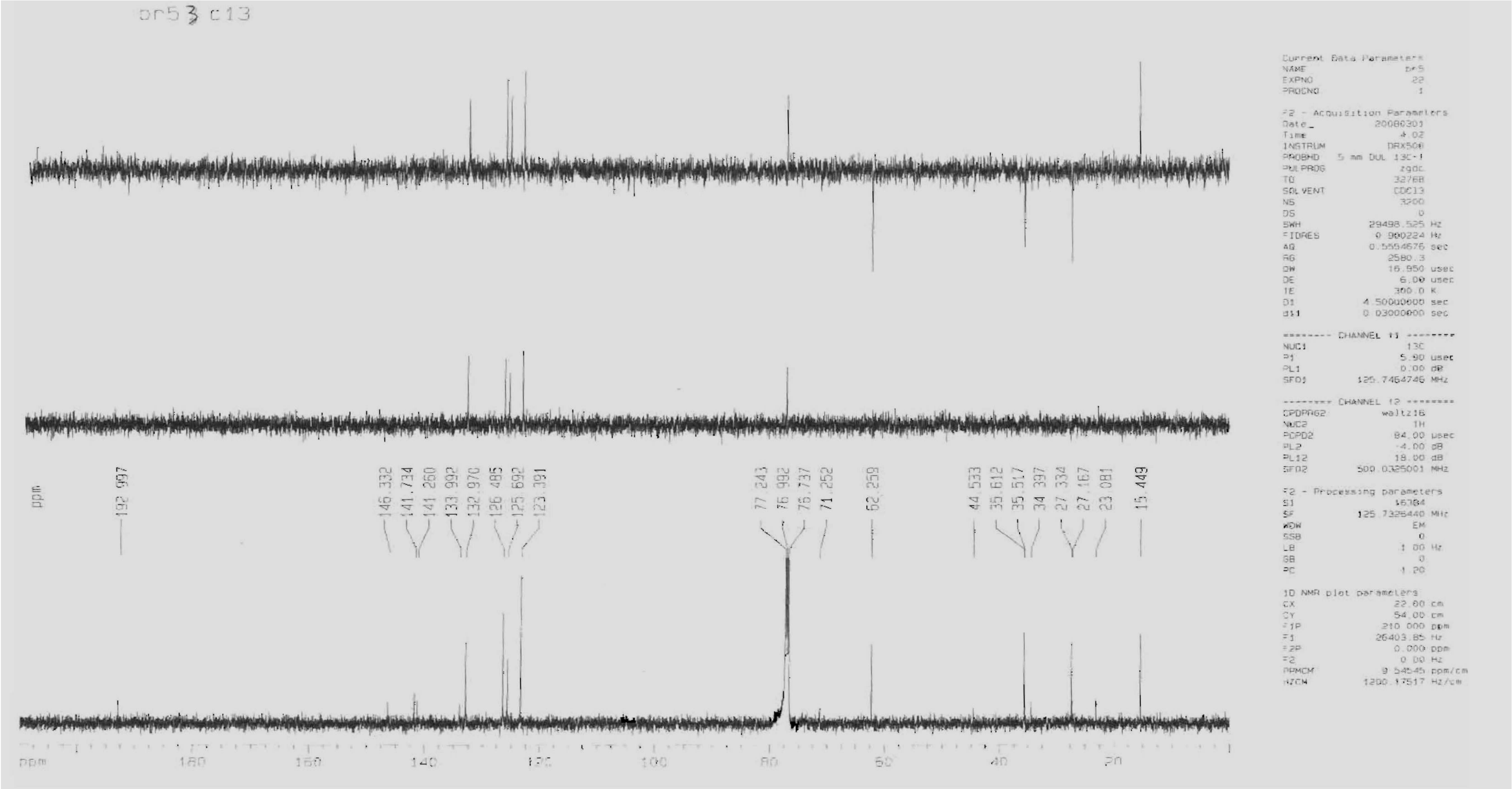

Figure 11 ^{13}C NMR (125 MHz, $CDCl_3$) of Compound **2**

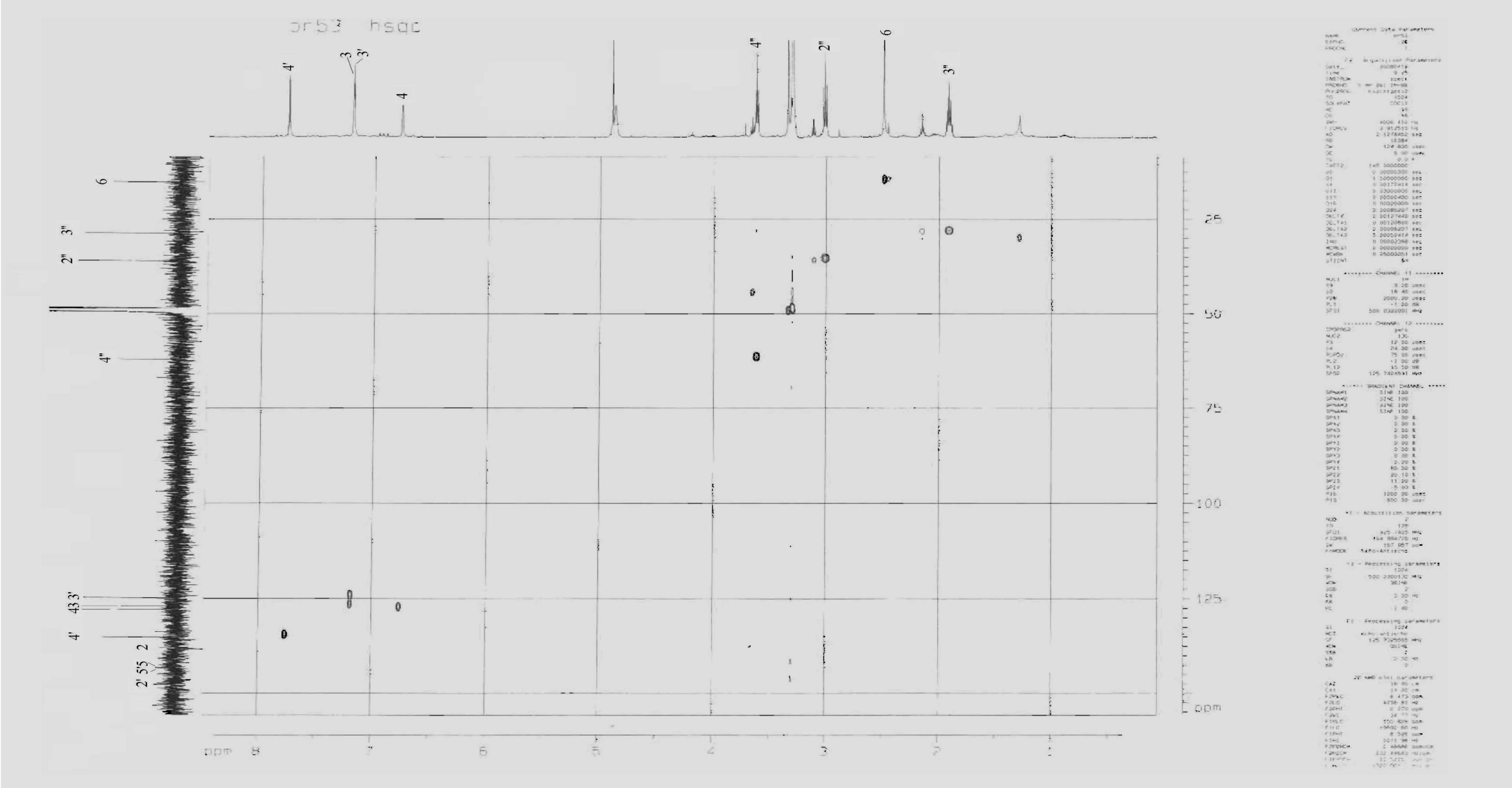

Figure 12 2D NMR (HSQC) of Compound **2**

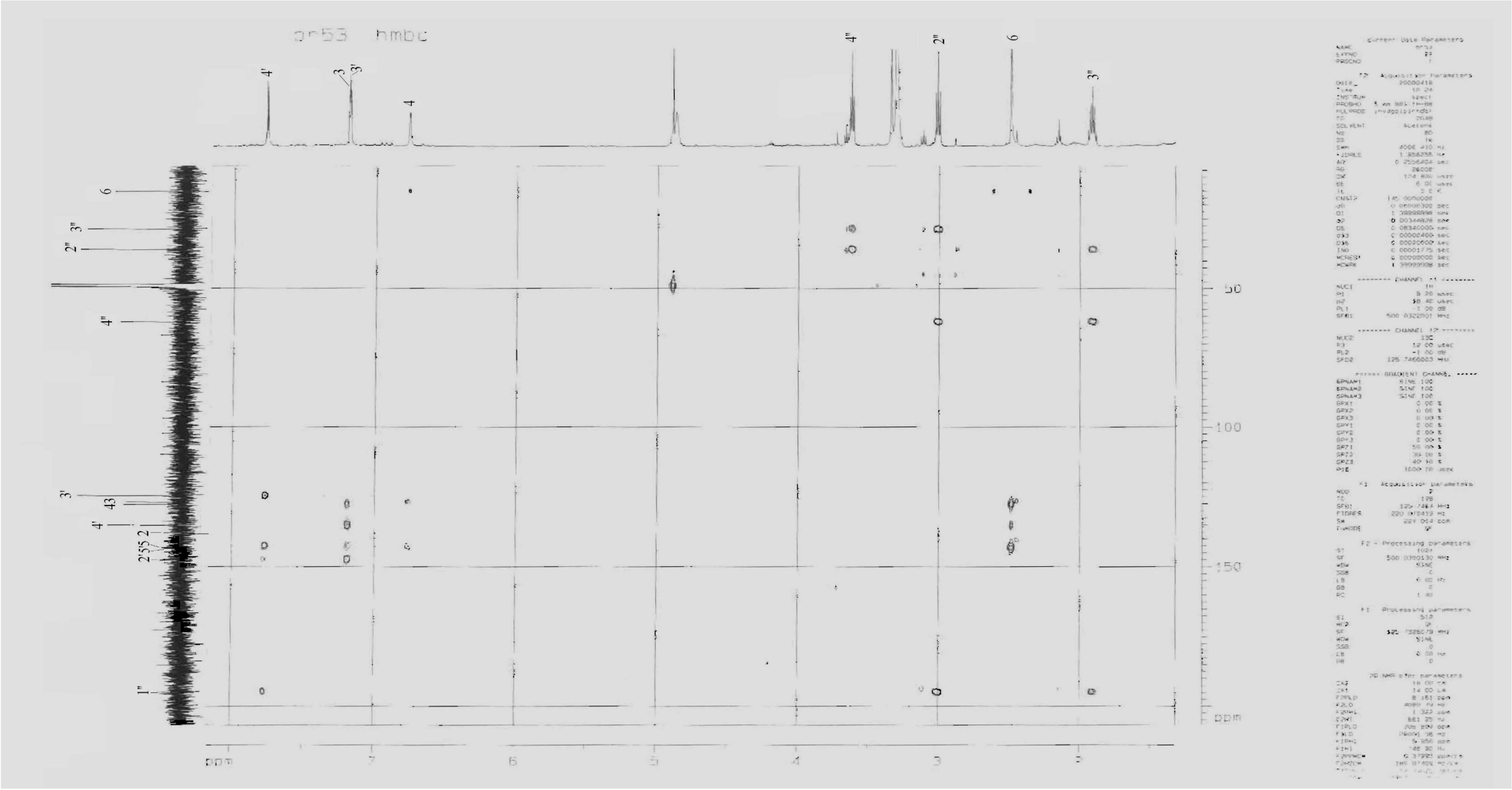

Figure 13 2D NMR (HMBC) of Compound **2**

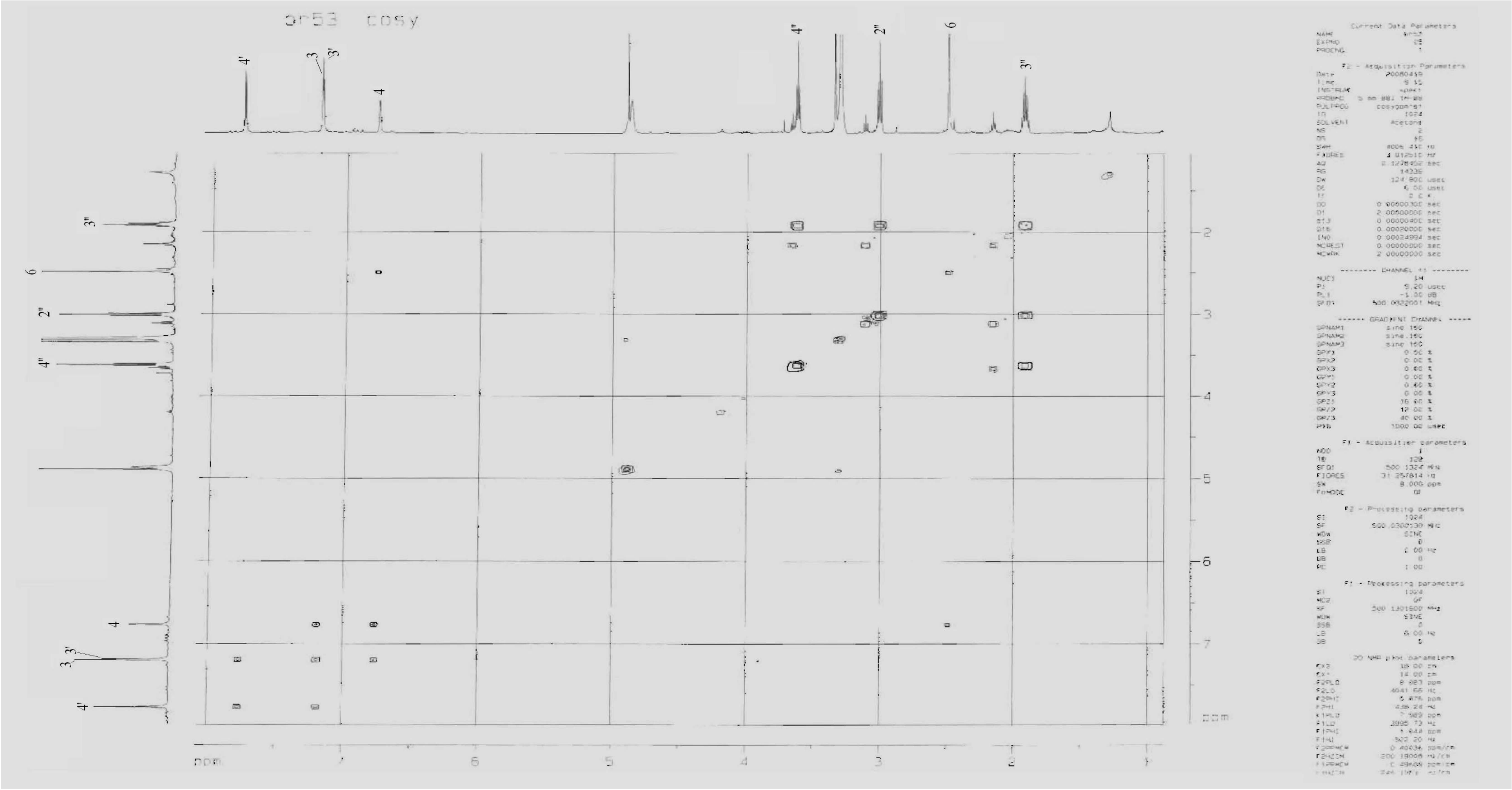

Figure 14 2D NMR (^1H-^1H COSY) of Compound **2**

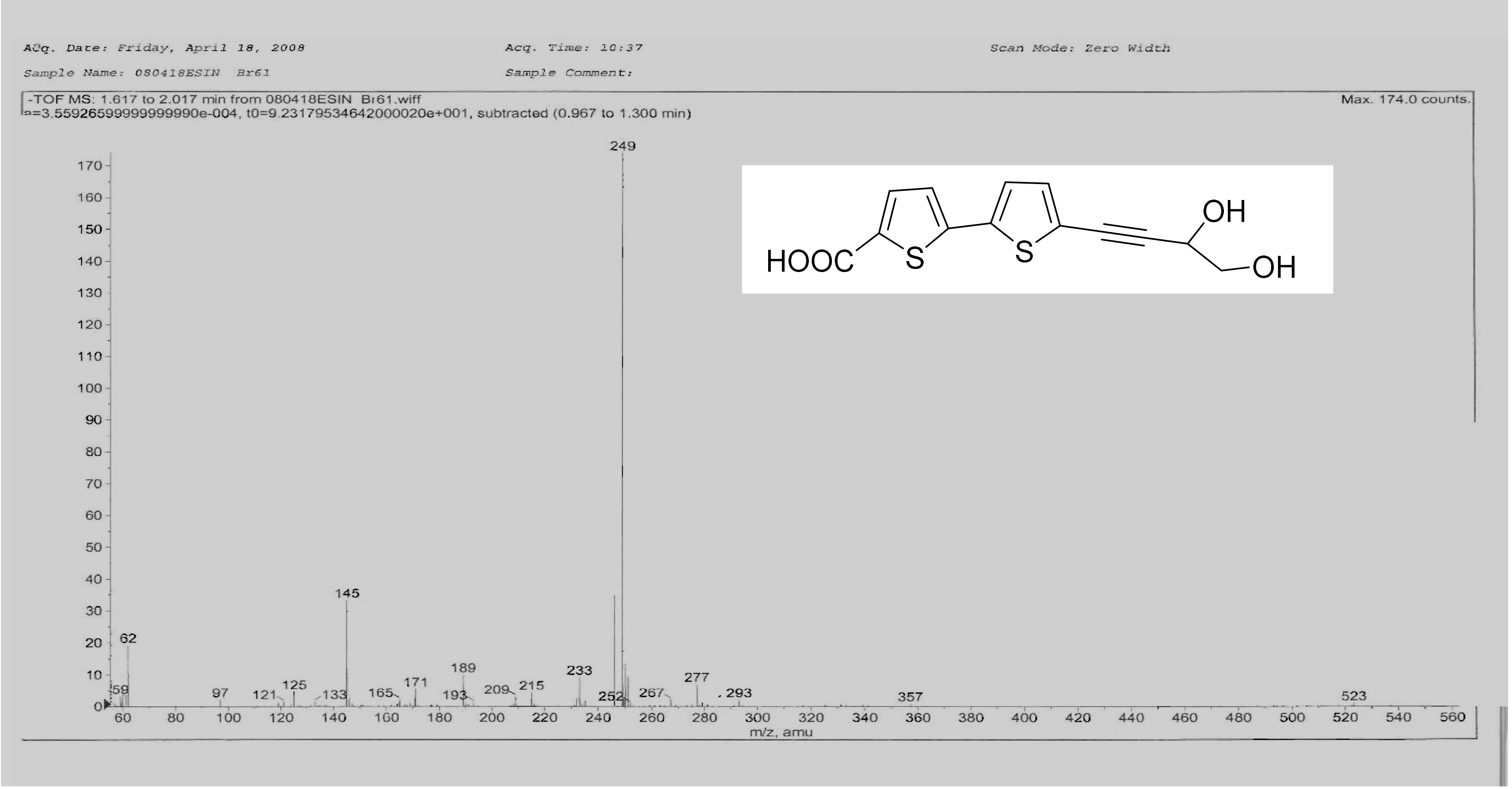
Acq. Date: Friday, April 18, 2008
Acq. Time: 10:37
Scan Mode: Zero Width
Sample Name: 080418ESIN Br61
Sample Comment:
-TOF MS: 1.617 to 2.017 min from 080418ESIN Br61.wiff
a=3.55926599999999990e-004, t0=9.23179534642000020e+001, subtracted (0.967 to 1.300 min)
Max. 174.0 counts.
249
145
62
59
97
121
125
133
165
171
189
193
209
215
233
252
267
277
293
357
523
m/z, amu
HOOC
S
S
OH
OH

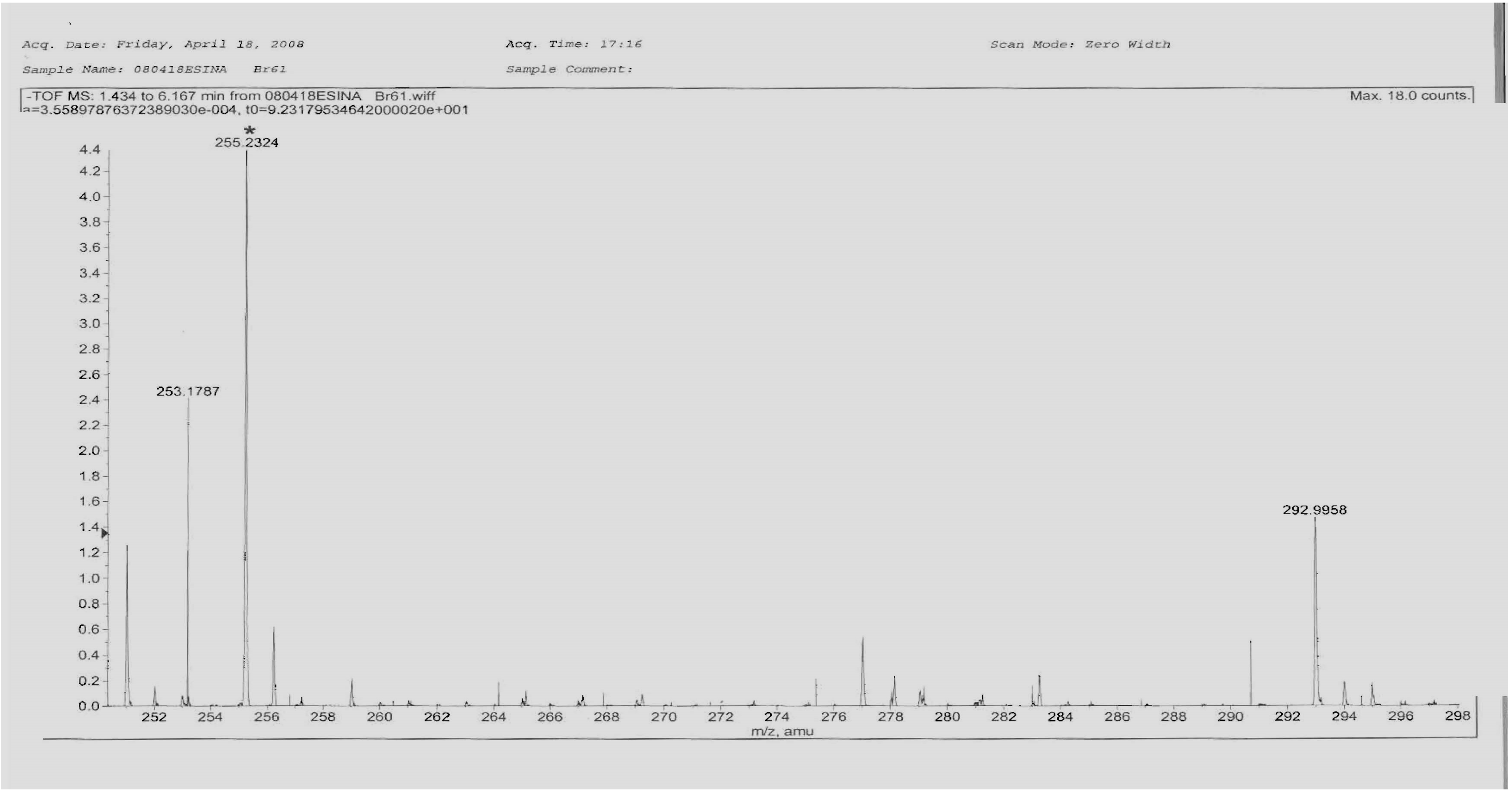
Acq. Date: Friday, April 18, 2008
Acq. Time: 17:16
Scan Mode: Zero Width
Sample Name: 080418ESINA Br61
Sample Comment:
-TOF MS: 1.434 to 6.167 min from 080418ESINA Br61.wiff
a=3.55897876372389030e-004, t0=9.23179534642000020e+001
Max. 18.0 counts.
255.2324
253.1787
292.9958
m/z, amu

Acq. Date: Friday, April 18, 2008 Acq. Time: 17:16 Scan Mode: Zero Width
Sample Name: 080418ESINA Br61 Sample Comment:

Elemental composition calculator

Target m/z: +292.9958 amu
Tolerance: +10.0000 ppm
Result type: Elemental
Max num of results: 1000
Min DBE: -5.0000 Max DBE: +60.0000
Electron state: OddAndEven
Num of charges: 0
Add water: N/A
Add proton: N/A
File Name: 080418ESINA Br61.wiff

	Elements	Min Number	Max Number
1	Br	0	0
2	C	0	200
3	Cl	0	0
4	F	0	0
5	H	0	400
6	I	0	0
7	N	0	0
8	Na	0	0
9	O	0	10
10	S	2	2

Acq. Date: Friday, April 18, 2008 Acq. Time: 17:16 Scan Mode: Zero Width
Sample Name: 080418ESINA Br61 Sample Comment:

	Formula	Calculated m/z (amu)	mDa Error	PPM Error	DBE
1	C13 H9 O4 S2	292.9942	1.5724	5.3669	9.5

Figure 15 HR-ESI-MS of Compound **3**

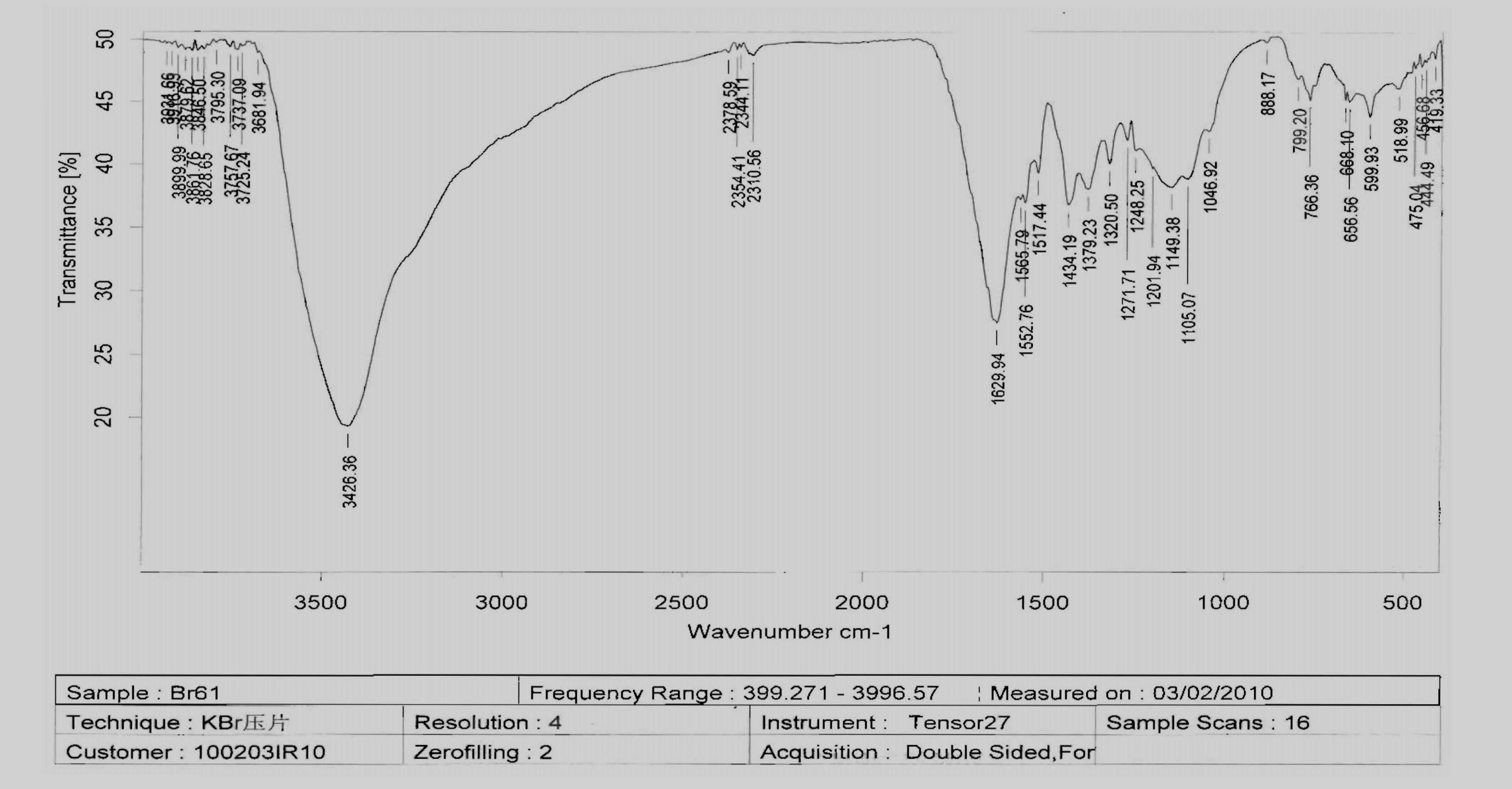

Figure 16 IR of Compound **3**

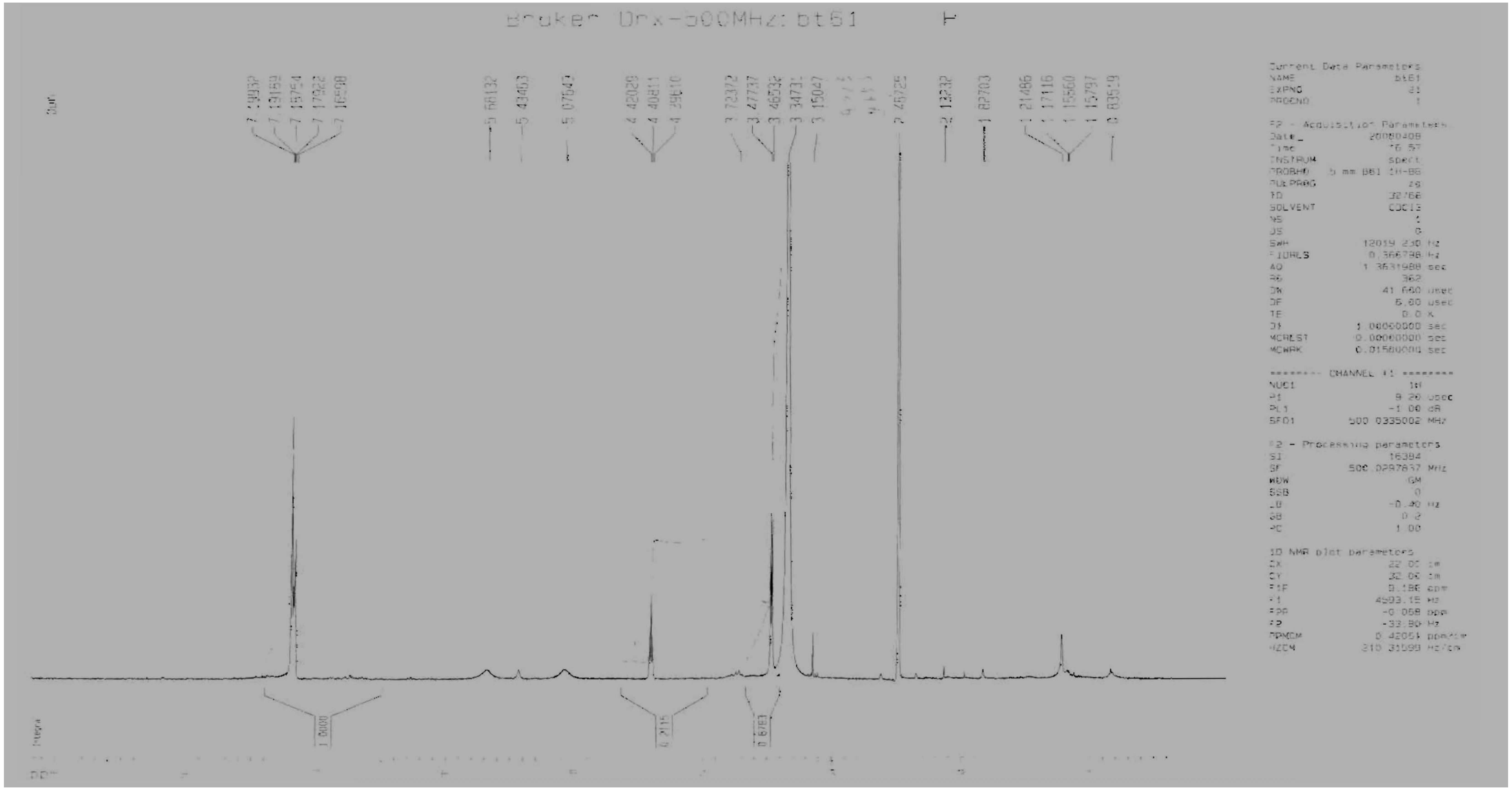

Figure 17 ^{1}H NMR (500 MHz, CD_3OD) of Compound **3**

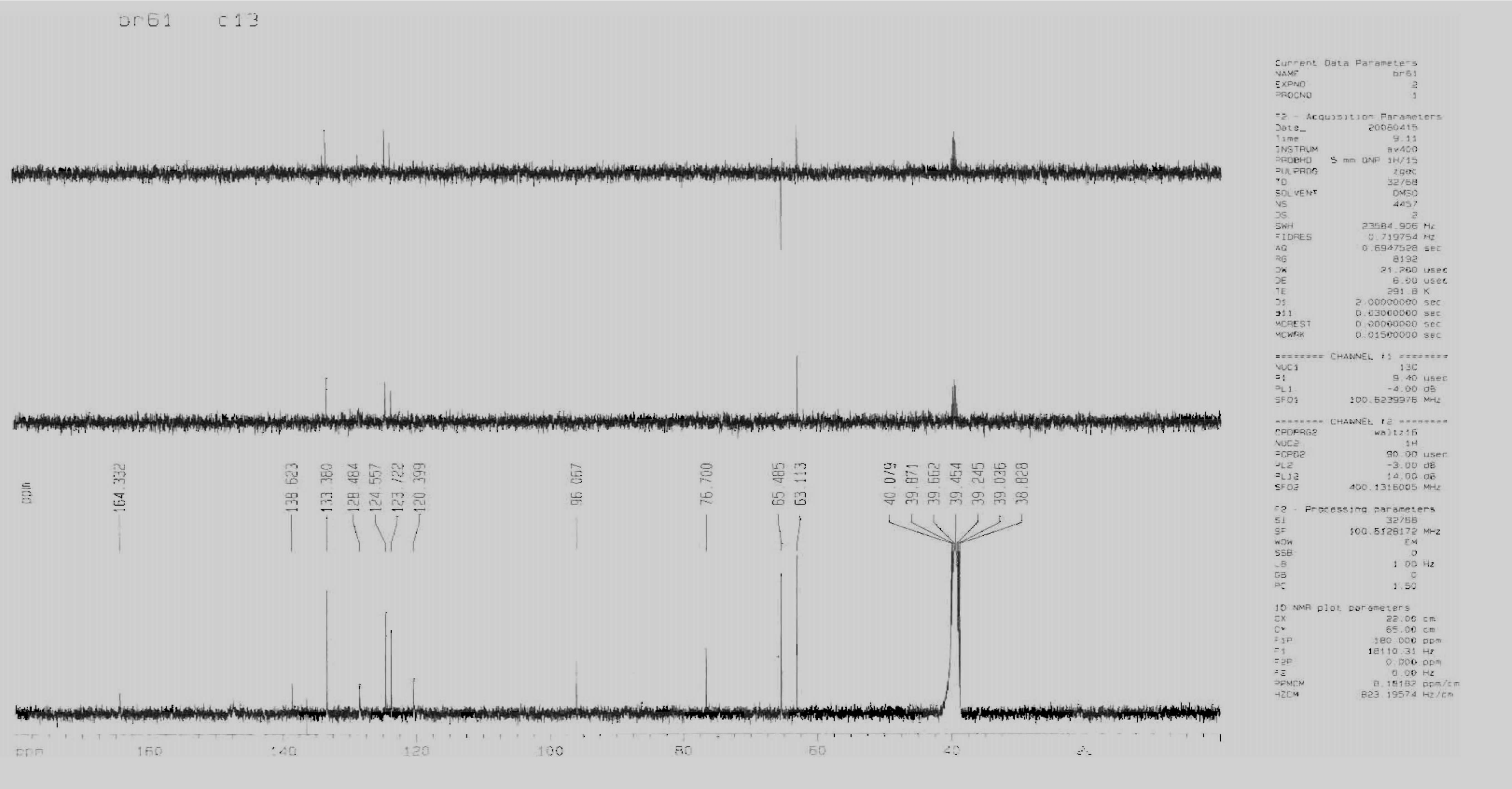

Figure 18 ^{13}C NMR (125 MHz, CD_3OD) of Compound **3**

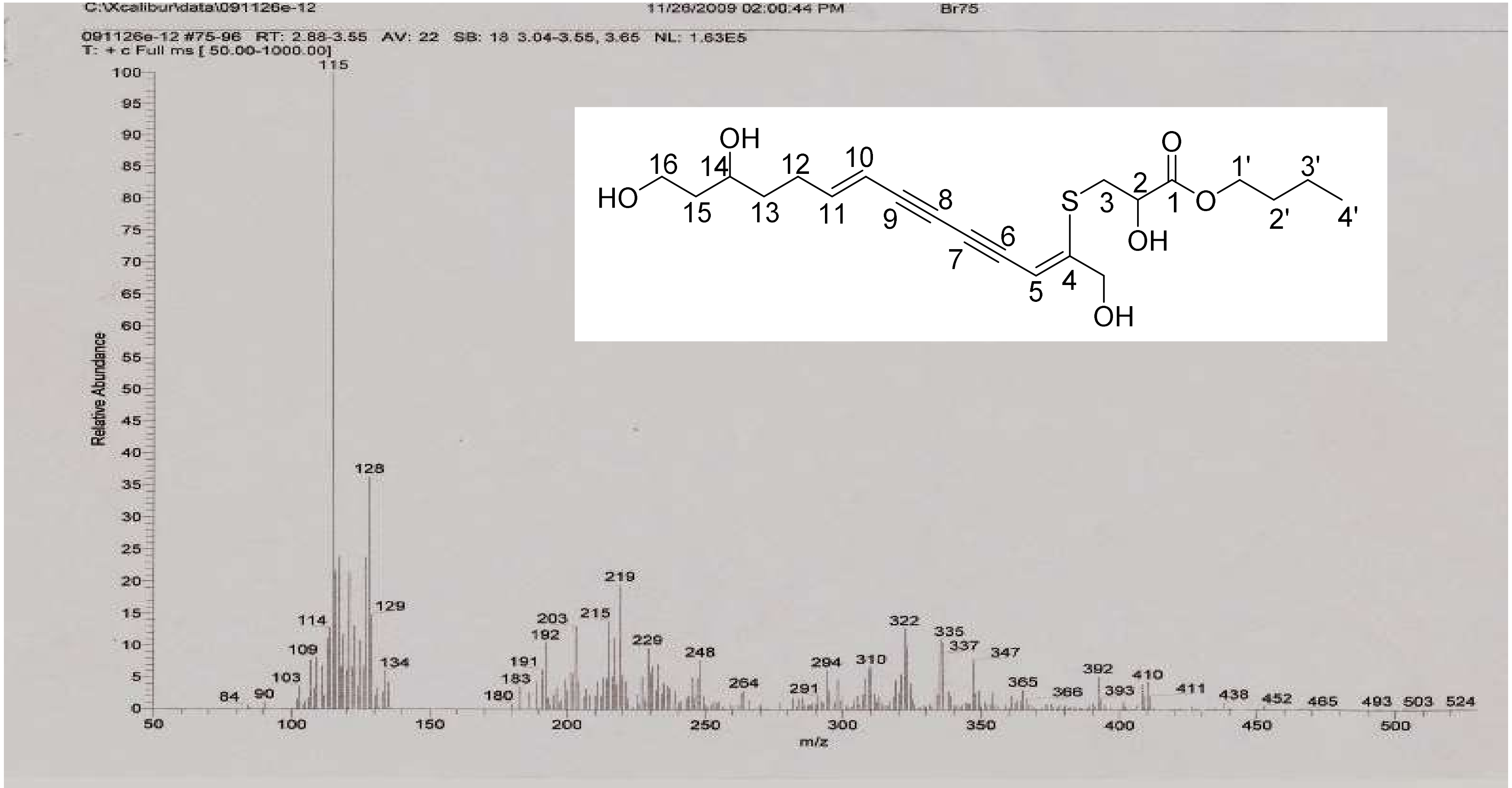
C:\Xcalibur\data\091126e-12
11/26/2009 02:00:44 PM
Br75
091126e-12 #75-96 RT: 2.88-3.55 AV: 22 SB: 18 3.04-3.55, 3.65 NL: 1.63E5
T: + c Full ms [50.00-1000.00]
Relative Abundance
m/z
115
128
129
134
114
109
103
90
84
219
203
215
192
191
183
180
229
248
264
291
294
310
322
335
337
347
365
366
392
393
410
411
438
452
465
493
503
524
HO
OH
OH
OH
O
O
S
16
14
12
10
15
13
11
9
8
7
6
5
4
3
2
1
1'
2'
3'
4'

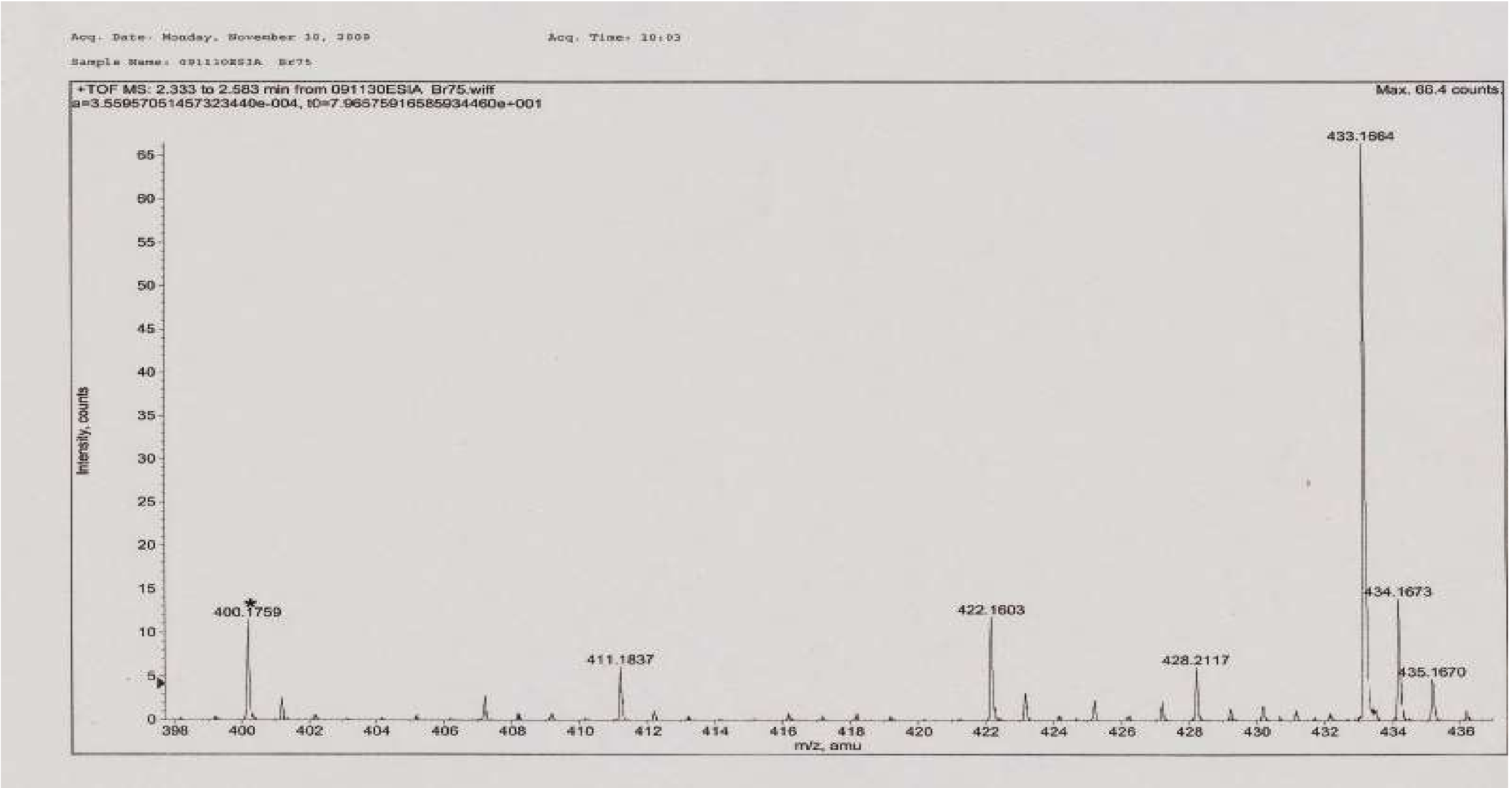

Figure 19 HR-ESI-MS of Compound **4**

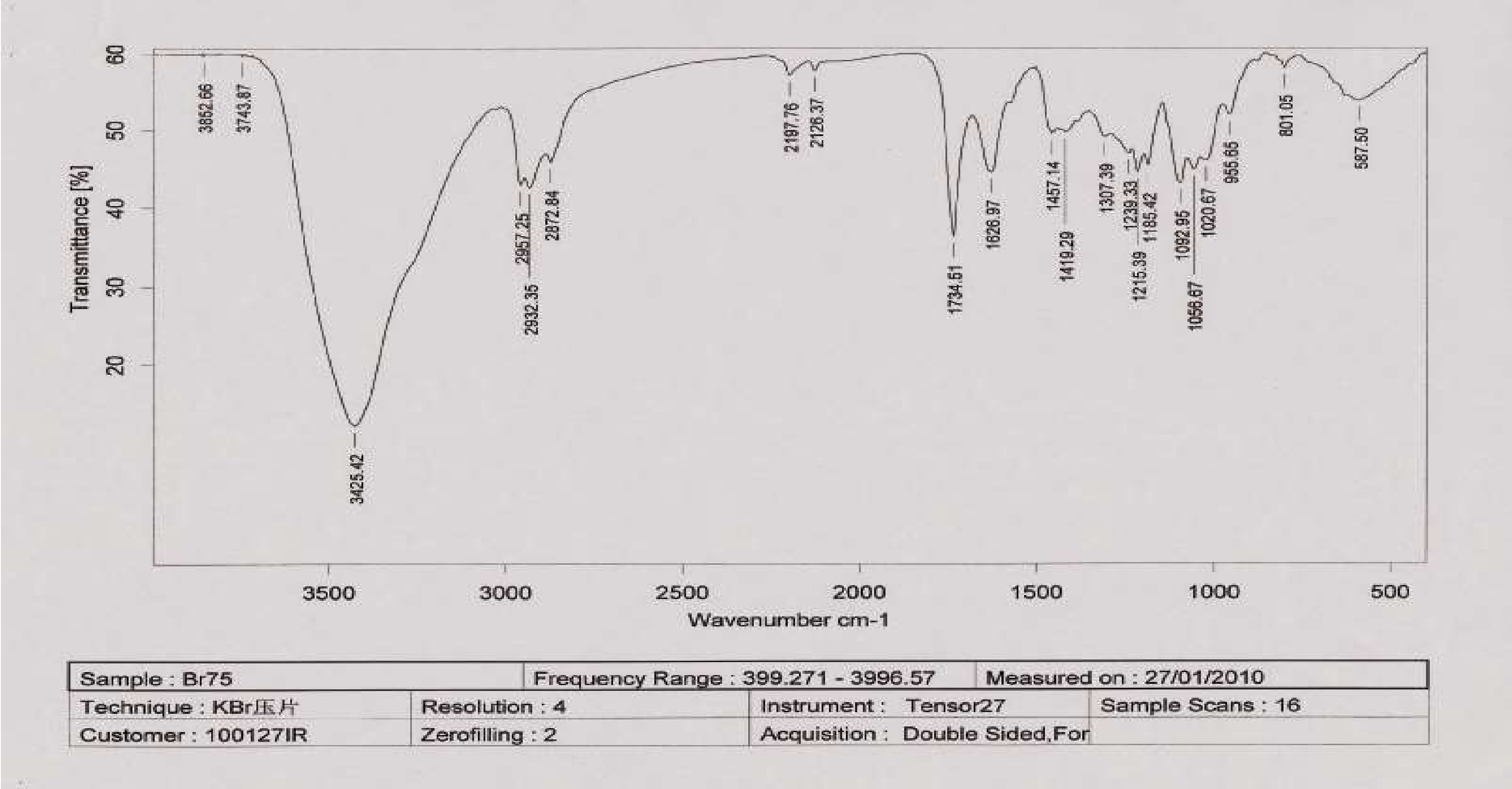

Figure 20 IR of Compound **4**

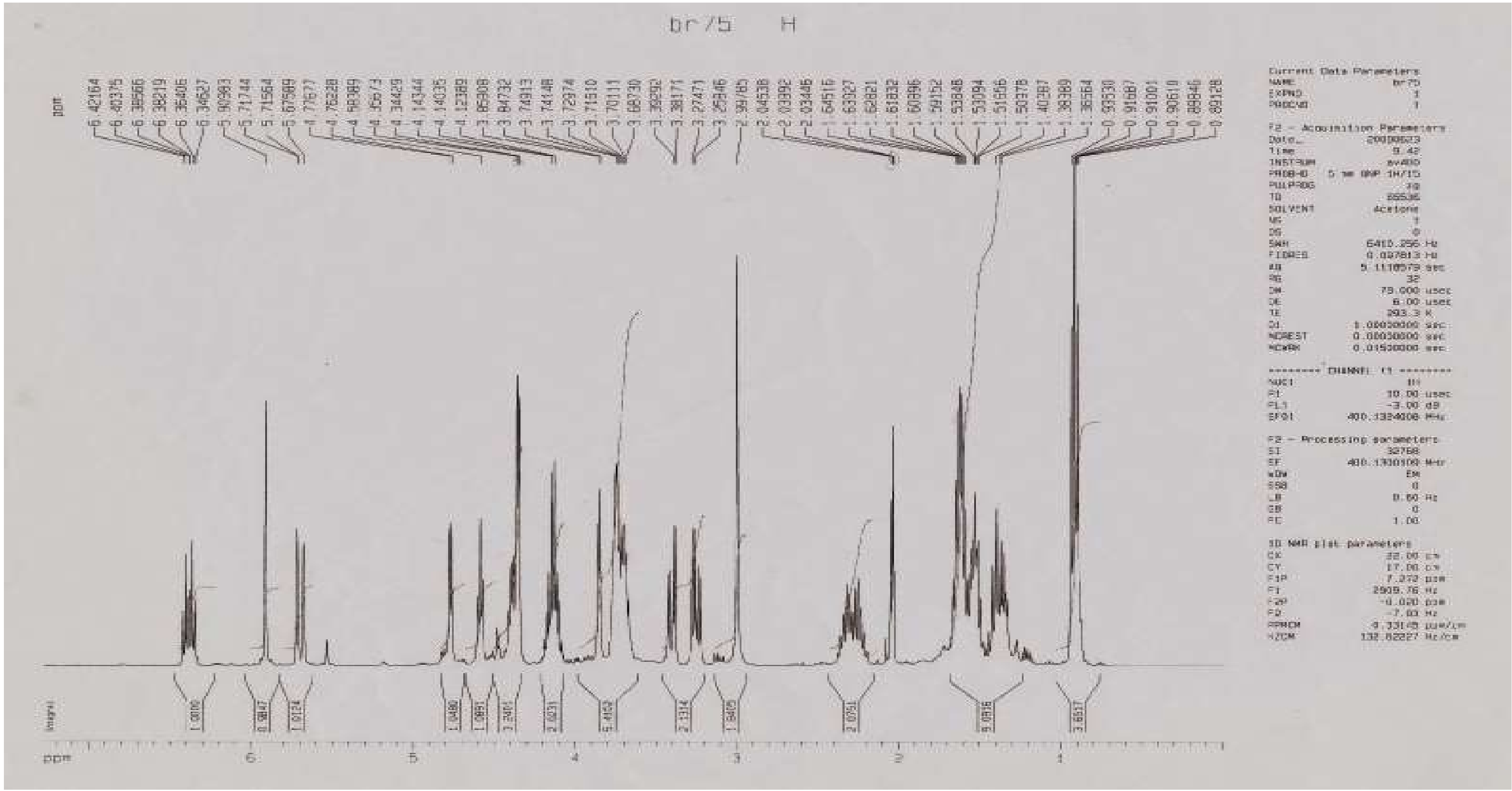

Figure 21 ^{1}H NMR (400 MHz, CD_3COCD_3) of Compound 4

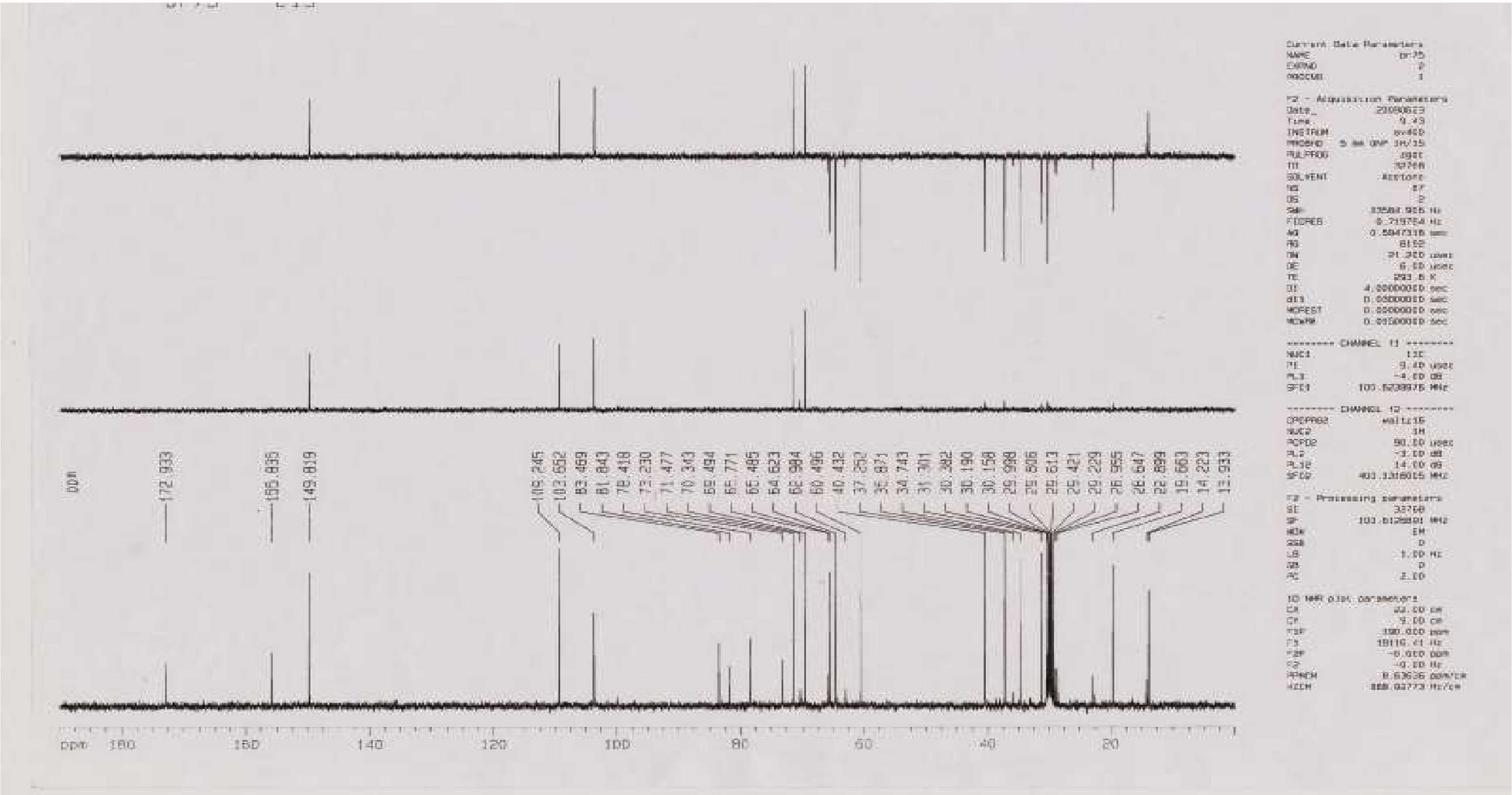

Figure 22 ^{13}C NMR (100 MHz, CD_3COCD_3) of Compound 4

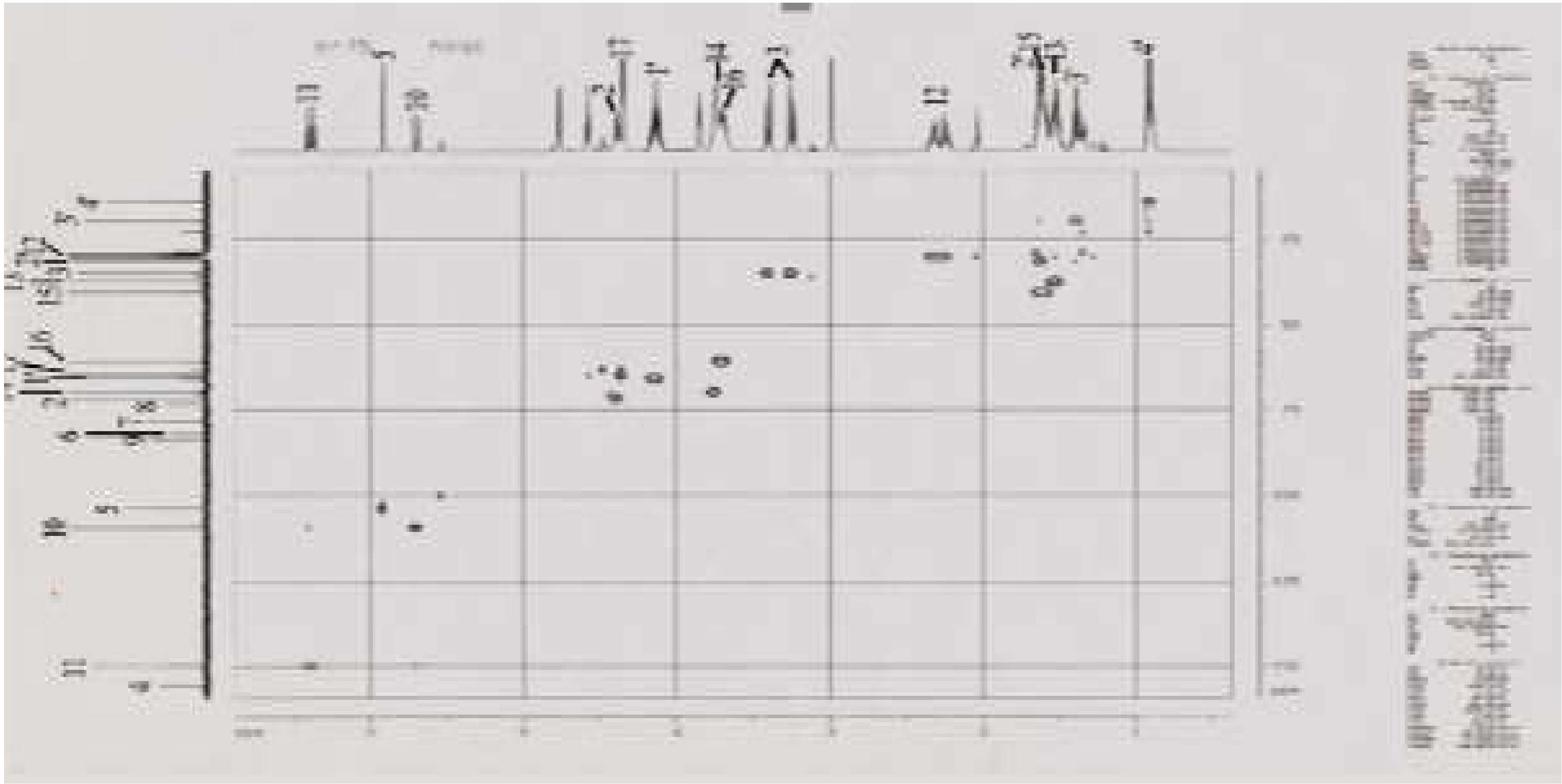

Figure 23 2D NMR (HSQC) of Compound **4**

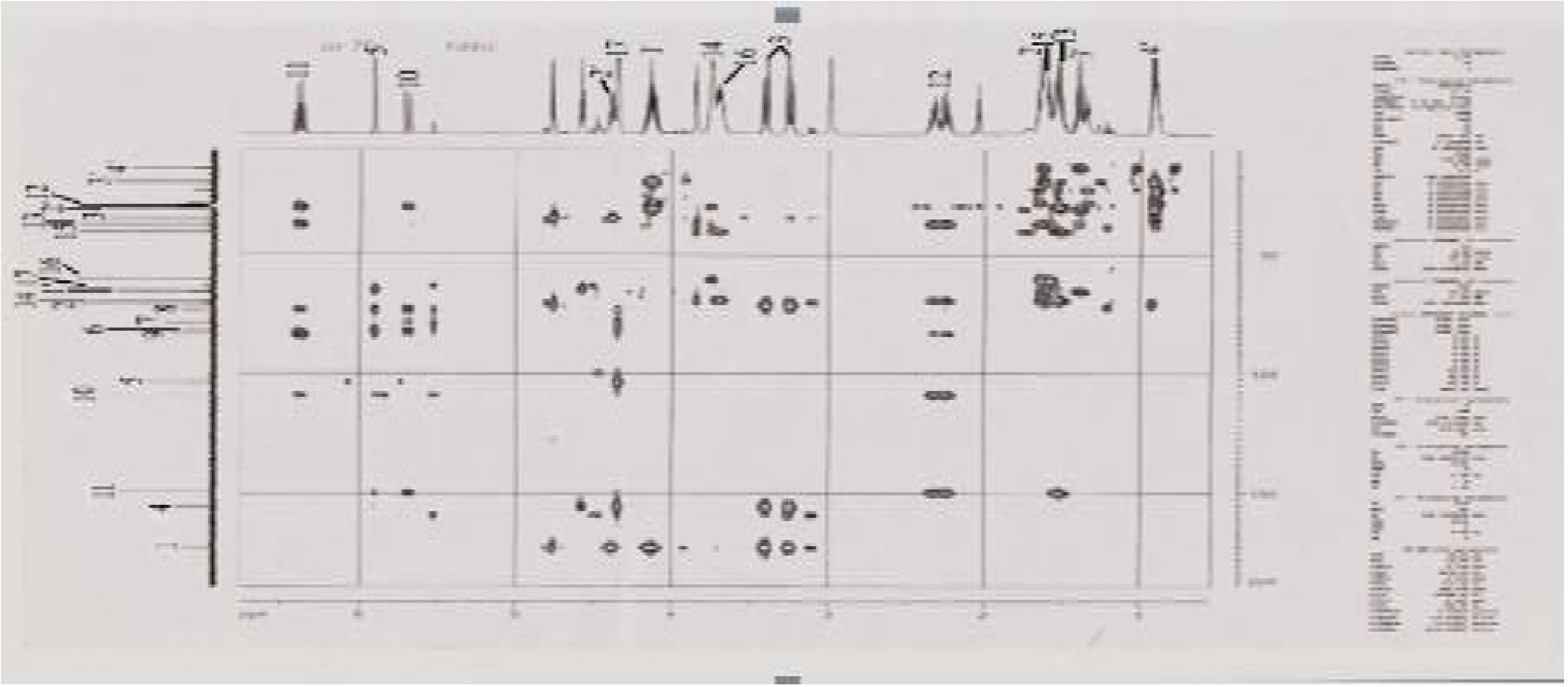

Figure 24 2D NMR (HMBC) of Compound **4**

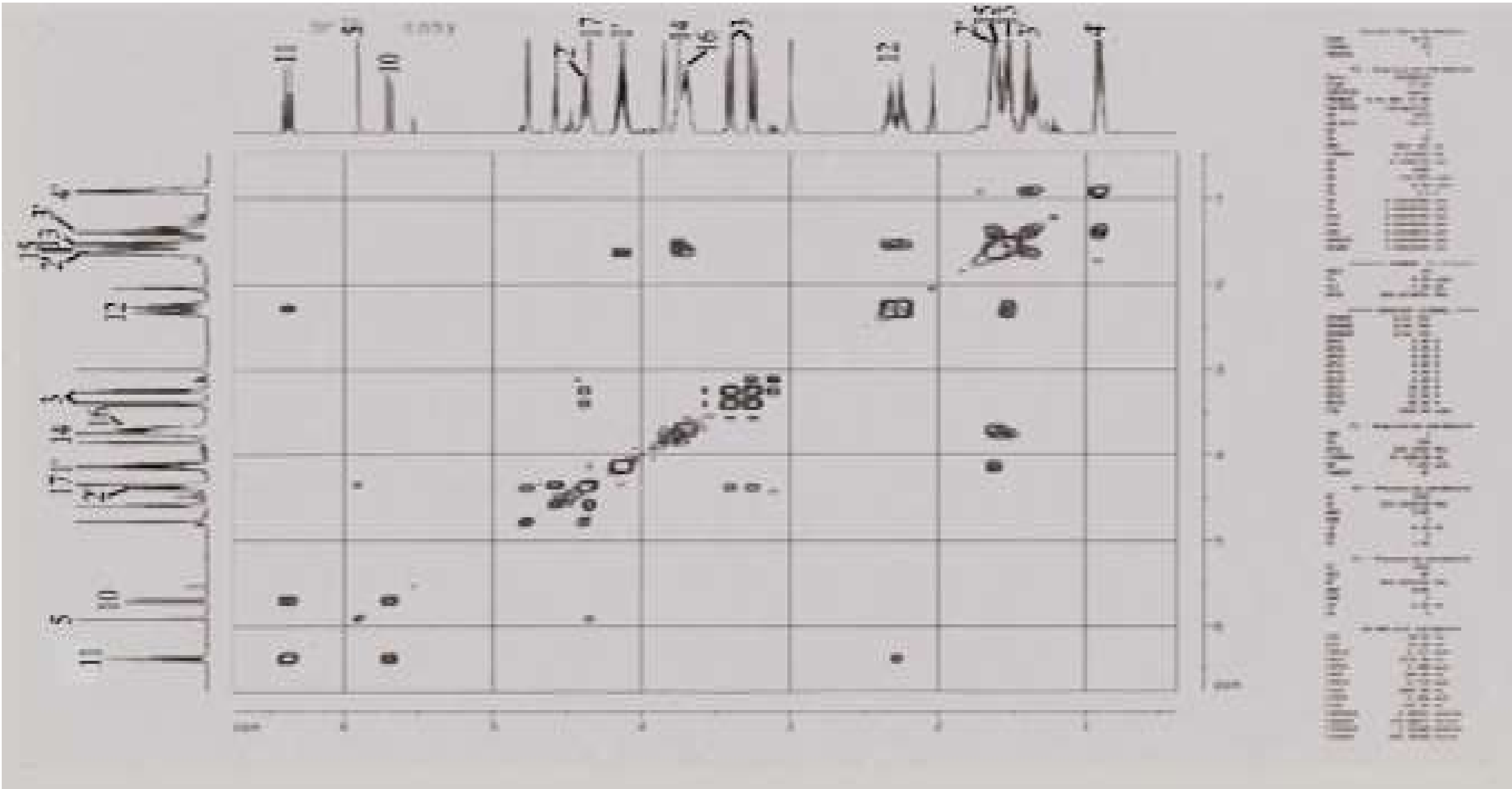

Figure 25 2D NMR (^1H-^1H COSY) of Compound **4**

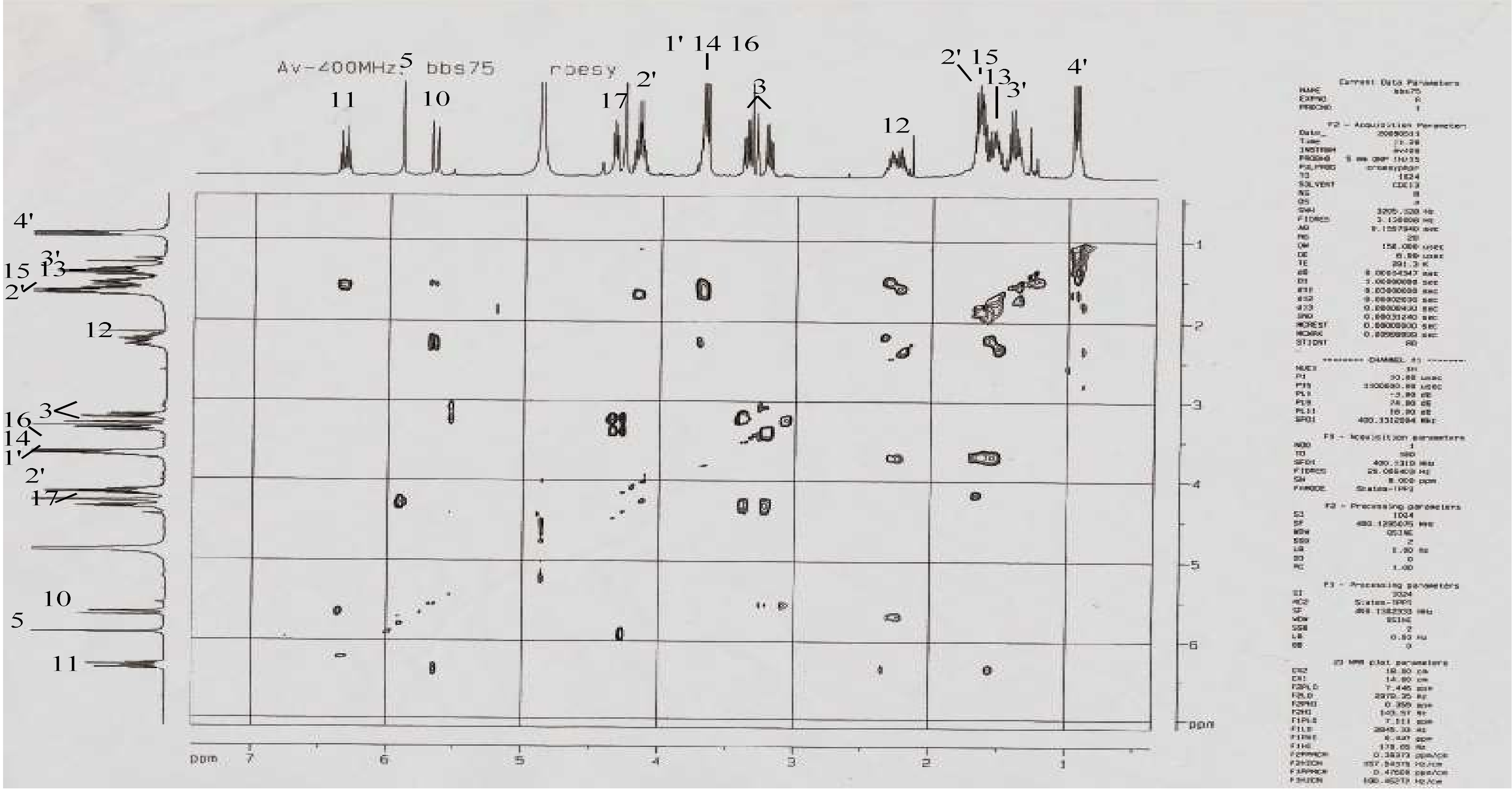

Figure 26 2D NMR (ROESY) of Compound **4**
